# Supplementary material for: Analyzing the fine structure of distributions
Source: PLoS One. 2020 Oct 14;15(10):e0238835. doi: 10.1371/journal.pone.0238835 (PMC7556505; doi:10.1371/journal.pone.0238835)
Supplement: S1 File — (DOCX) [file pone.0238835.s001.docx]

**S1 File: ITS and MTY**

**
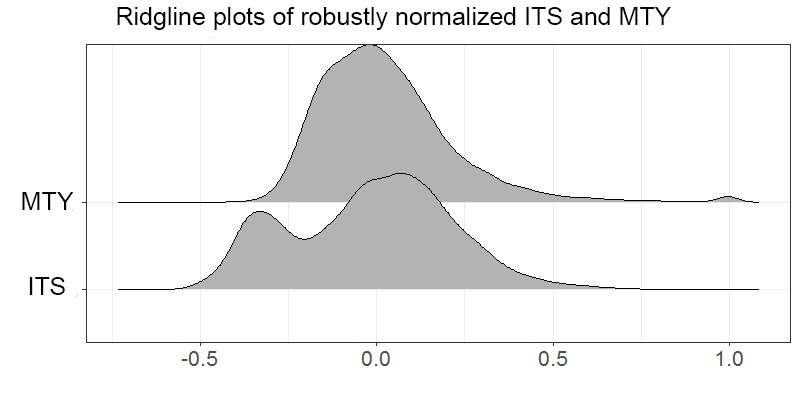
**


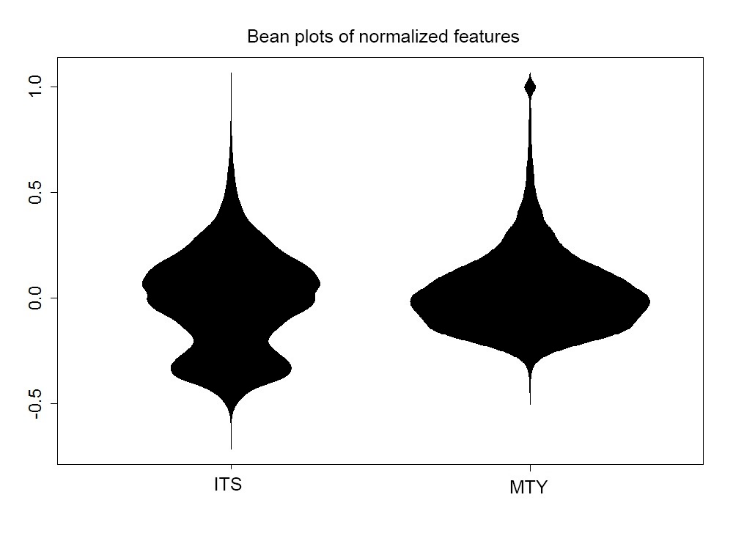


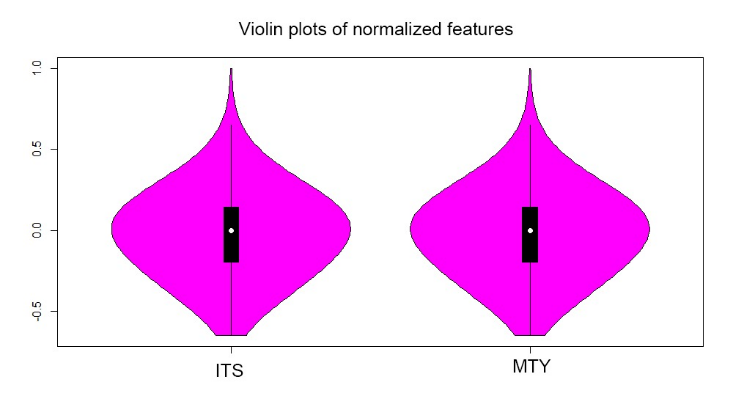


Fig A: Visualization of the distribution of two normalized features of the MD plot with a bean plot (left, b) a violin plot (right, c) and a ridgeline plot (top, a). The violin plot is unable to show bimodality. The bean plot shows two to three modes for the ITS feature.
