# Supplementary material for: Analyzing the fine structure of distributions
Source: PLoS One. 2020 Oct 14;15(10):e0238835. doi: 10.1371/journal.pone.0238835 (PMC7556505; doi:10.1371/journal.pone.0238835)
Supplement: S2 File — (DOCX) [file pone.0238835.s002.docx]

**S2 File: Descriptive Statistics**

Table A: Descriptive statistics of selected features from German companies noted on the stock market reporting by the Prime standard using the R package ‘fBasics’ on CRAN [61]. The ordering of the features is by concavity and the same as in Fig. 9 and 10 but from top to bottom instead of left to right. Six features from the bottom do not possess more than 1% negative values. A total of 50% of the data for net tangible assets and total cash flow from operating activities lies in a small positive range. Interest expense and capital expenditures do not possess more than 1% positive values. These results overlap with the MD plot in Fig. 9 but not with the bean plot in Fig. 10.

Abbreviations: M: Missing values, Q01: 1% quantile, Q99: 99% Quantile, 1.Qua: 1^st^ Quartile, 3.Qua: 3^rd^ Quartile, Ske: Skewness, Kurt: Kurtosis.

| Features | M | Q01 | Q99 | 1.Qua | 3.Qua | Mean | Median | Ske | Kurt |
| --- | --- | --- | --- | --- | --- | --- | --- | --- | --- |
| Net Income | 3 | -6.2E+04 | 2.3E+06 | **54** | 4.6E+04 | 1.4E+05 | 7.4E+03 | 4.7 | **24** |
| Treasury Stock | 28 | -3.6E+06 | 1.5E+07 | -2.2E+03 | 4.7E+05 | 9.8E+05 | 2.9E+04 | 6.8 | **66** |
| Net Tangible Assets | 0 | -4.4E+06 | 5.5E+07 | **1.4E+04** | **8.3E+05** | 2.1E+06 | 1.3E+05 | 4.7 | 26 |
| Total Cash Flow From Operating Activities | 26 | -1.1E+06 | 3.4E+06 | **-5.4E+03** | **5.1E+04** | 8.3E+04 | 4.5E+03 | 0.05 | 31 |
| Interest Expense | 19 | -3.9E+05 | **-3.5** | -1.3E+04 | -240 | -2.5E+04 | -1.9E+03 | **-5.1** | 30 |
| Capital Expenditures | 41 | -1.8E+06 | **-9.9** | -4.0E+04 | -1.1E+03 | -9.7E+04 | -6.3E+03 | **-5.1** | 29 |
| Total Revenue | 4 | **1.5E+03** | 3.1E+07 | 4.3E+04 | 8.7E+05 | 2.1E+06 | 1.7E+05 | 5.8 | 40 |
| Gross Profit | 4 | **59** | 7.4E+06 | 1.8E+04 | 3.1E+05 | 5.4E+05 | 6.7E+04 | 4.3 | 22 |
| Total Operating Expenses | 3 | **2.7E+03** | 2.8E+07 | 3.5E+04 | 8.1E+05 | 1.9E+06 | 1.5E+05 | 5.9 | 41 |
| Total Assets | 10 | **2.4E+04** | 4.5E+08 | 2.3E+05 | 6.6E+06 | 2.6E+07 | 1.2E+06 | 8.9 | 93 |
| Total Liabilities | 10 | 7.0E+03 | 3.7E+08 | 9.7E+04 | 4.1E+06 | 2.1E+07 | 6.2E+05 | 9.6 | 110 |
| Total Stockholder Equity | 0 | **1.7E+03** | 6.6E+07 | 9.0E+04 | 2.30+06 | 4.4E+06 | 4.3E+05 | 5.3 | 32 |

**References**

61. Wuertz D, Setz T, Chalabi Y. fBasics: Rmetrics - Markets and Basic Statistics. CRAN; 2017.
