# Supplementary material for: Analyzing the fine structure of distributions
Source: PLoS One. 2020 Oct 14;15(10):e0238835. doi: 10.1371/journal.pone.0238835 (PMC7556505; doi:10.1371/journal.pone.0238835)
Supplement: S3 File — (DOCX) [file pone.0238835.s003.docx]

**S3 File: Conventional Violin plot in Python**

The violin plots shown in this section were created with the Python package ‘seaborn’ (17), and the default value (Scott's rule of thumb) of the bandwidth parameter was used.


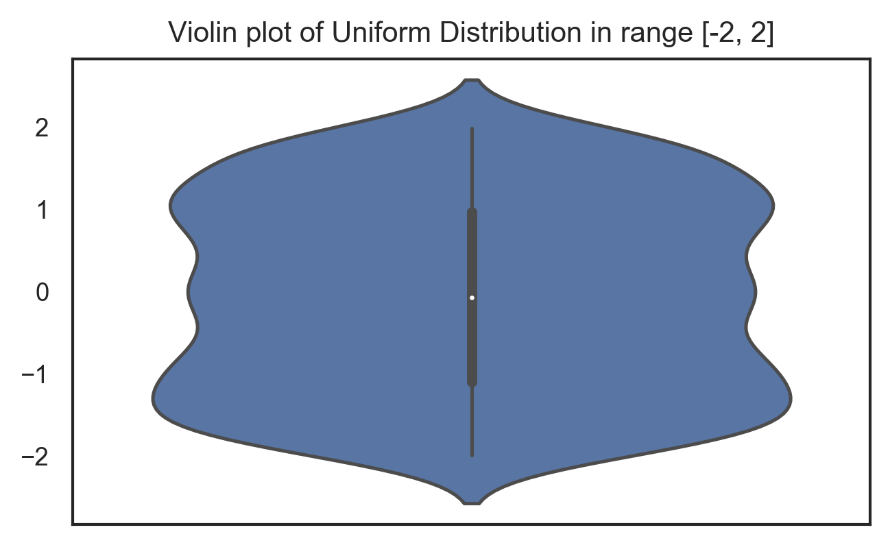


Fig A: Uniformly distributed data visualized as violin plots in Python. The violin plot suggests multimodality, while the MD plot shows the correct uniform distribution.


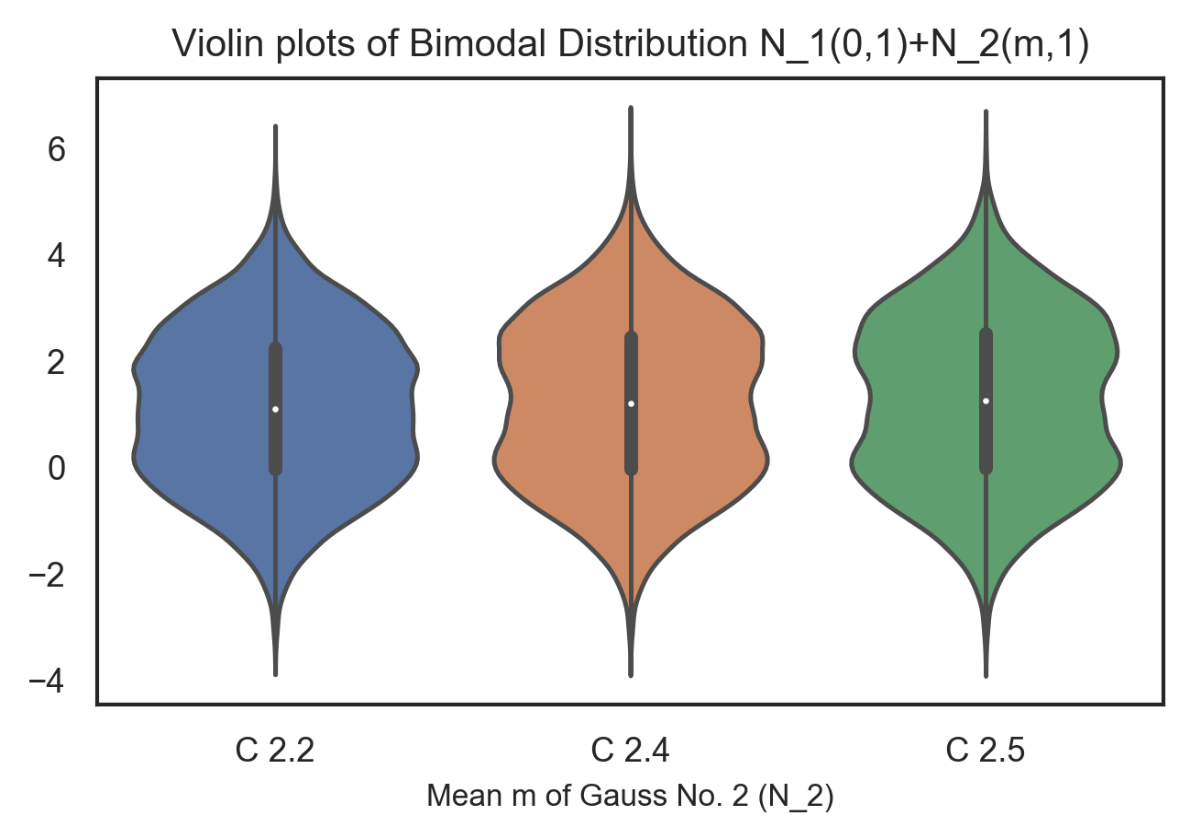


Fig B: Data with a bimodal distribution visualized as a violin plot in Python. Similar to the MD plot, the violin plot shows the bimodality of these data.


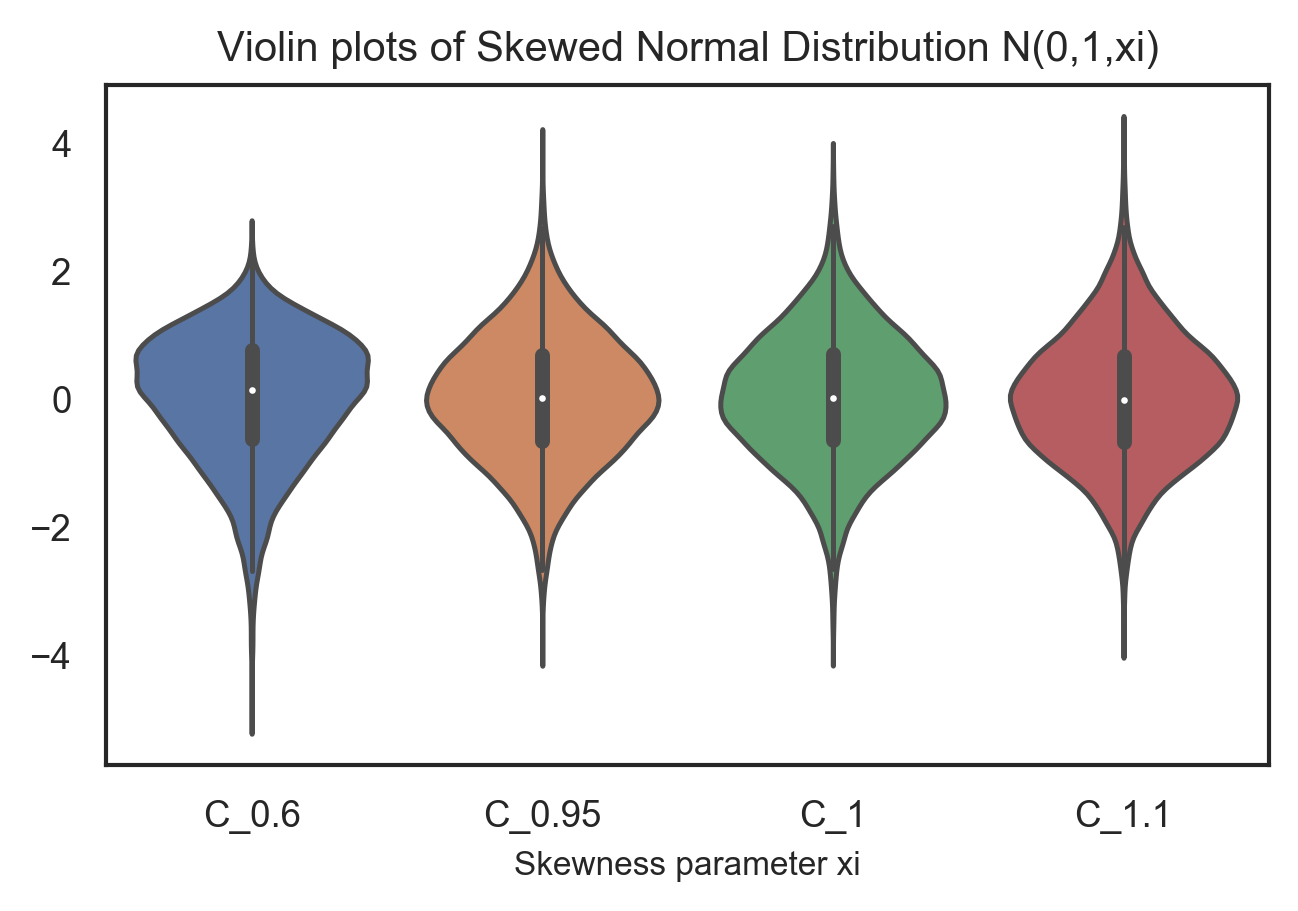


Fig C: The skewness of these unimodal distributions is visible in this violin plot, but this plot is slightly less sensitive than in the MD plot.


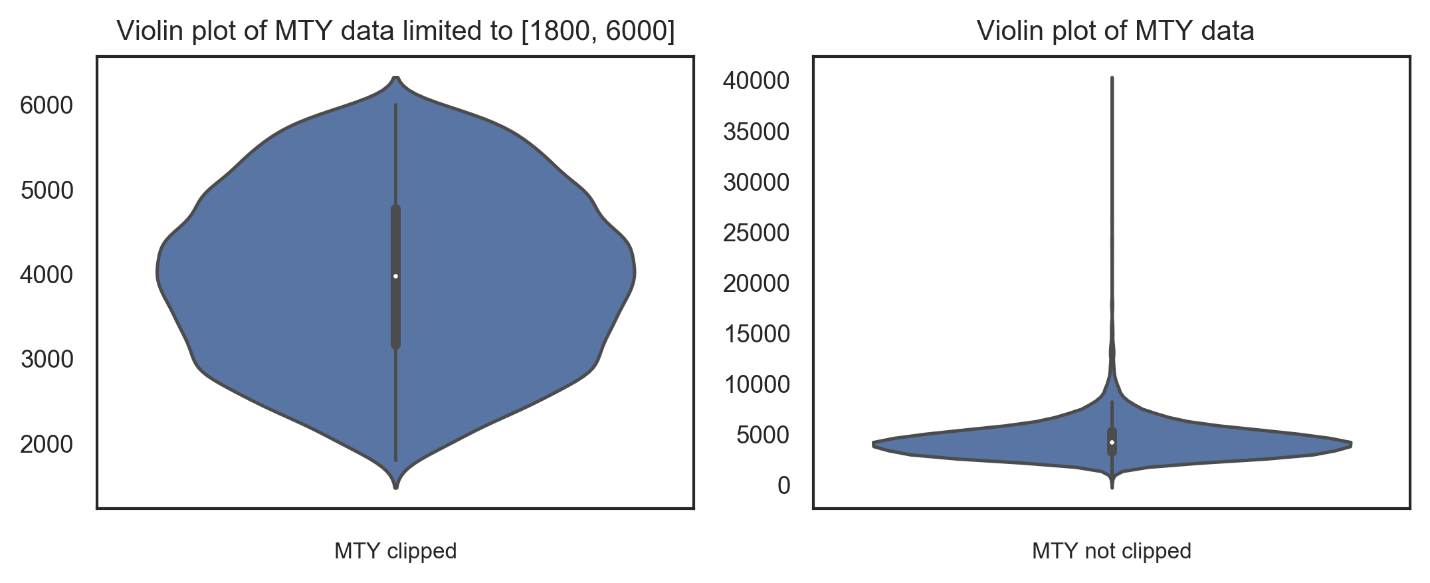


Fig D: The data for the left visualization were limited to the range [1800, 6000]. Nevertheless, in contrast to the MD plot, the violin plot goes beyond this range.


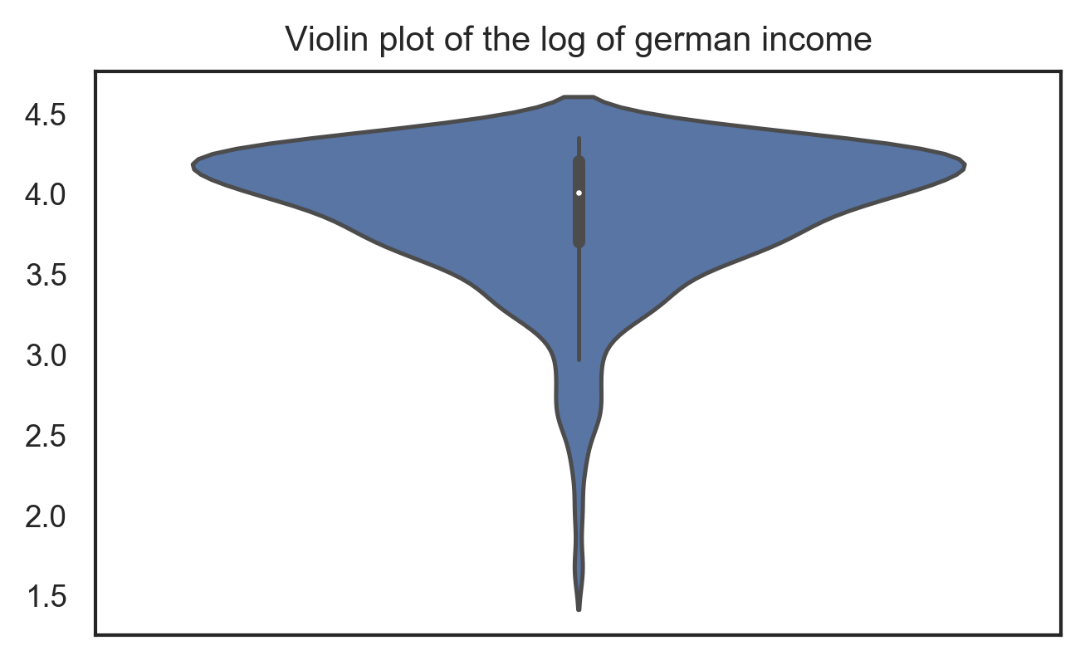


Fig E: Visualization of the log of German income. The violin plot shows values above 4.35 and a less detailed, smoother distribution than the MD plot.


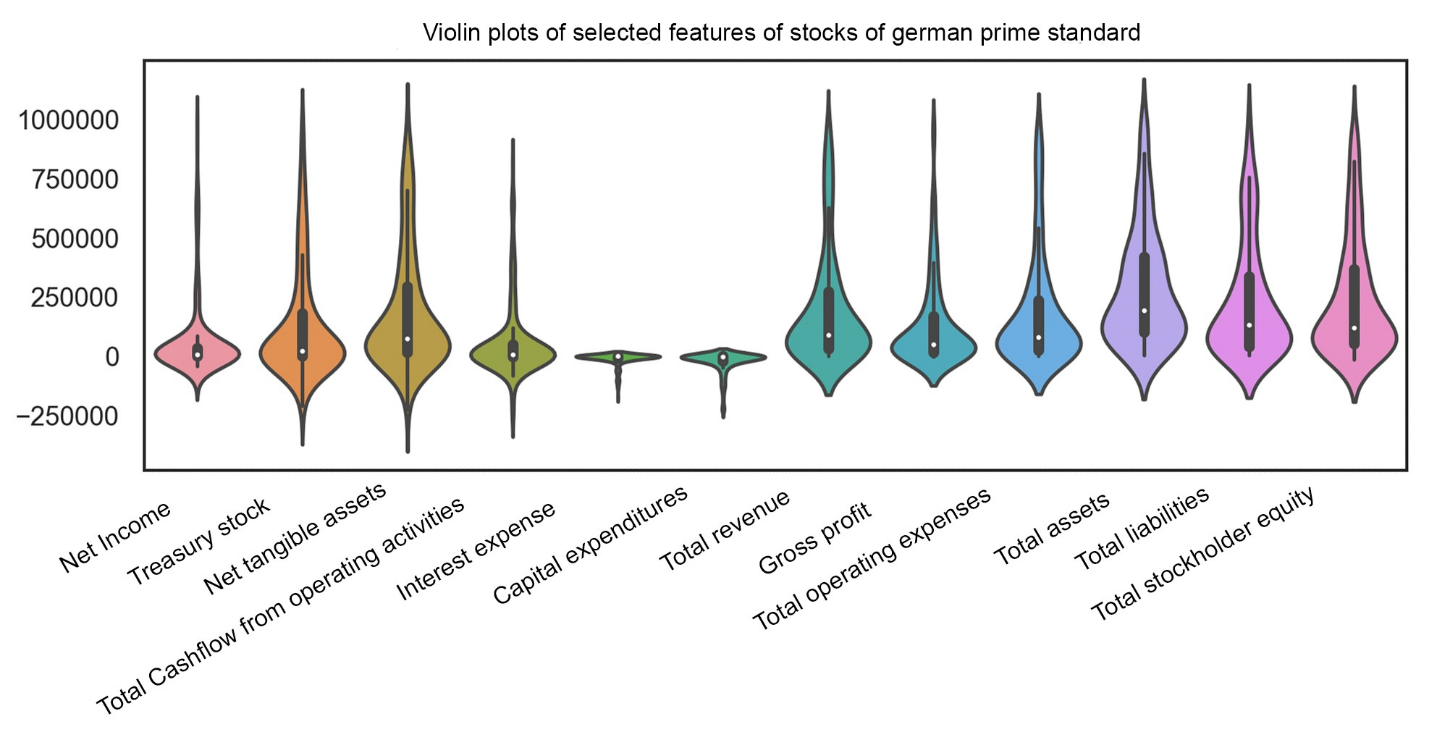


Fig F: Visualization of selected features from 269 companies on the German stock market reporting quarterly financial statements by the Prime standard. The violin plot shows data above and below the limits [-250000, 1000000] and a less detailed, more smoothed distribution than the MD plot.
