# Supplementary material for: Analyzing the fine structure of distributions
Source: PLoS One. 2020 Oct 14;15(10):e0238835. doi: 10.1371/journal.pone.0238835 (PMC7556505; doi:10.1371/journal.pone.0238835)
Supplement: S4 File — (DOCX) [file pone.0238835.s004.docx]

**S4 File: Overlayed histograms**

Each histogram is computed separately and thereafter integrated in one plot using plotly in R (23).


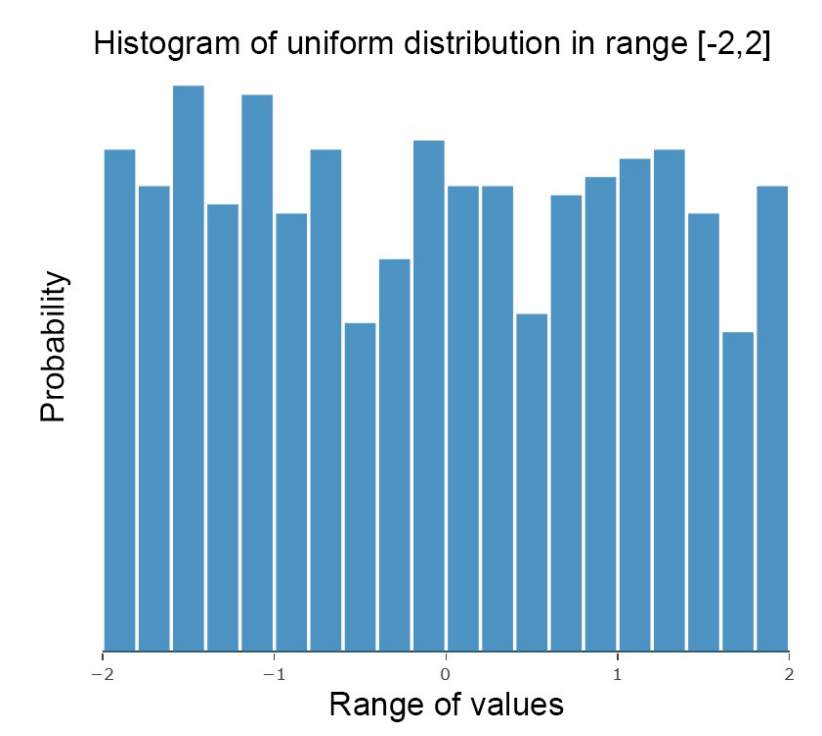


Fig A: Uniform distribution in the interval $[-2,2]$ of a 1000-point sample visualized by a histogram of plotly [32] with a default binwidth of plotly does not indicate a uniform distribution.


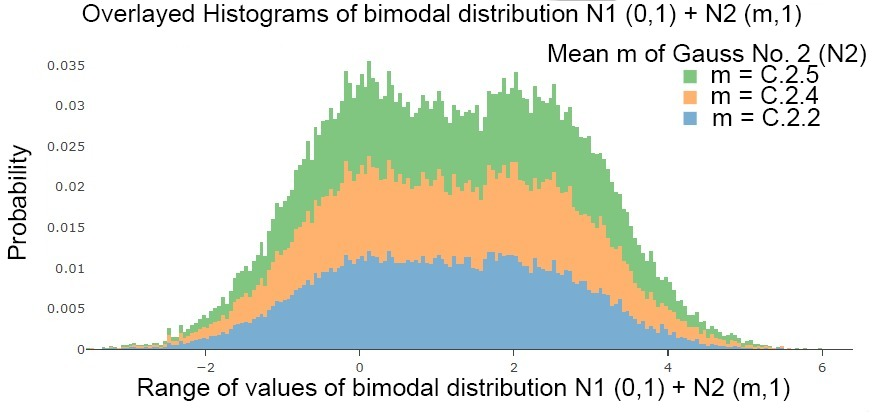


Fig B: Histograms with a default binwidth in plotly [32] are less sensitive than statistical testing, bean plots or MD plots in the case of bimodality. The setting of the parameter for the bin width in the plotly is currently not documented in R.


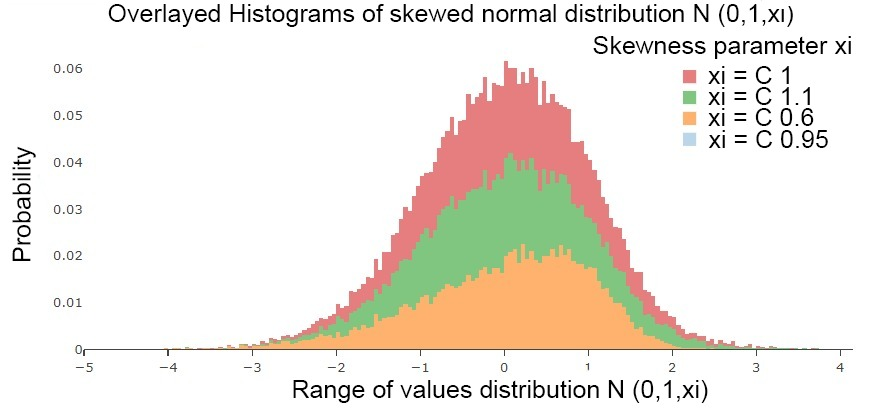


Fig C: Histograms with a default binwidth in plotly [32] are less sensitive than the MD plot for the skewness of the distribution.


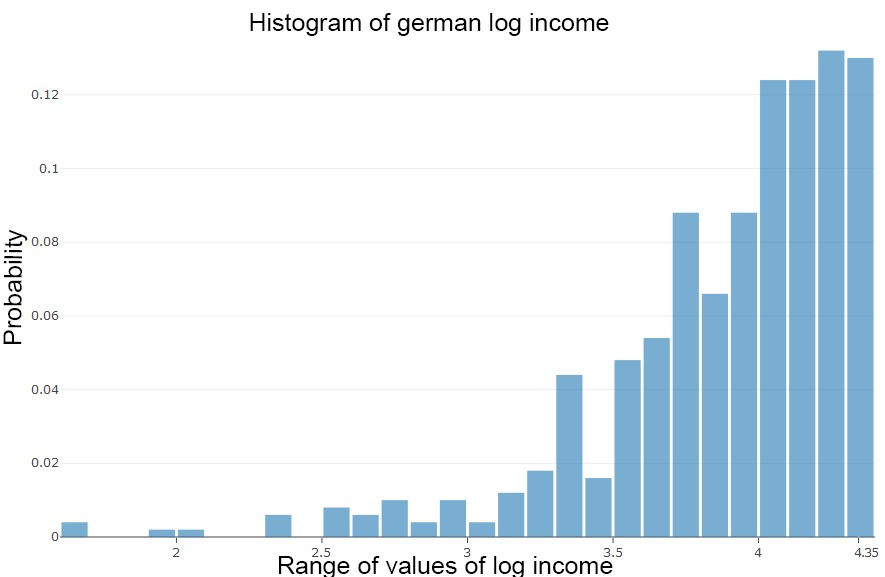


Fig D: Distribution analyses performed on the log of German people’s income in 2003 with a histogram of plotly [32] with a default binwidth.


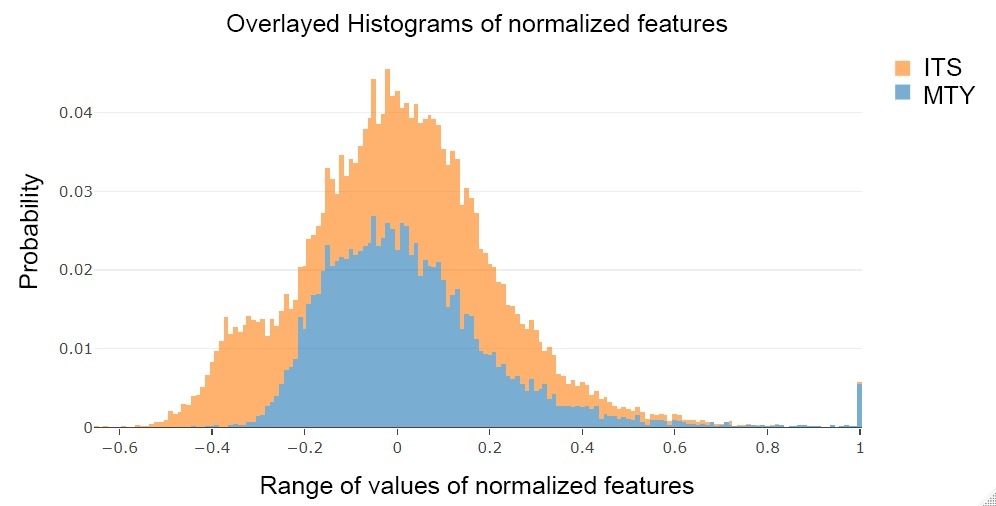


Fig E: Visualization of the distribution of two normalized features of the MD plot with an overlayed histogram of plotly [32] with a default binwidth. The overlayed histogram shows the bimodal distribution less clearly than the MD plot or bean plot.
