# Supplementary material for: Analyzing the fine structure of distributions
Source: PLoS One. 2020 Oct 14;15(10):e0238835. doi: 10.1371/journal.pone.0238835 (PMC7556505; doi:10.1371/journal.pone.0238835)
Supplement: S5 File — (DOCX) [file pone.0238835.s005.docx]

**S5 File: Density and ridgeline plots in Python**

This section covers density plots and ridgeline plots created by using the ‘kdeplot’ function of the ‘seaborn’ package. The default value (Scott's rule of thumb) of the bandwidth parameter was used.


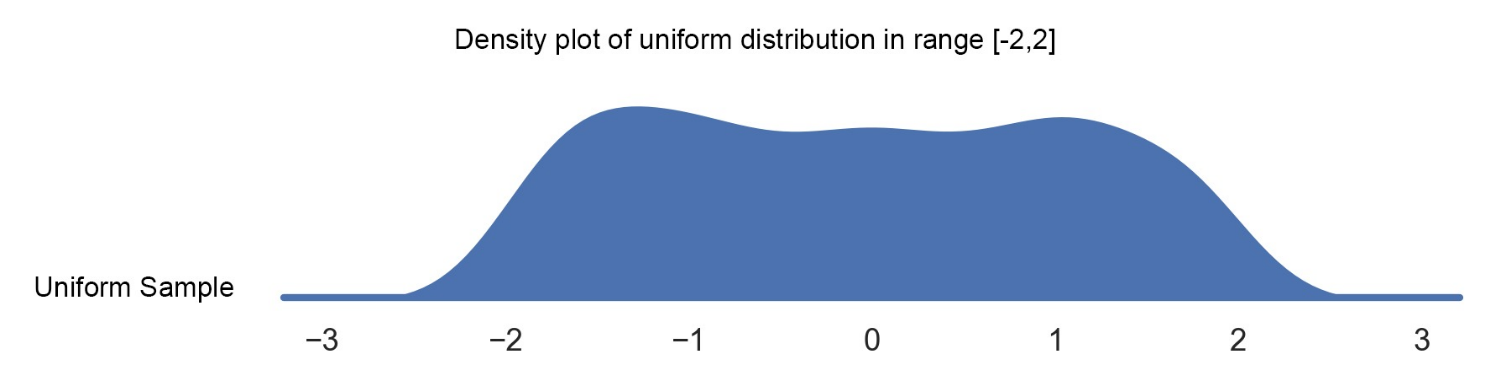


Fig A: Uniformly distributed data visualized as a density plot in Python. The density plot suggests multimodality, while the MD plot shows the correct uniform distribution.


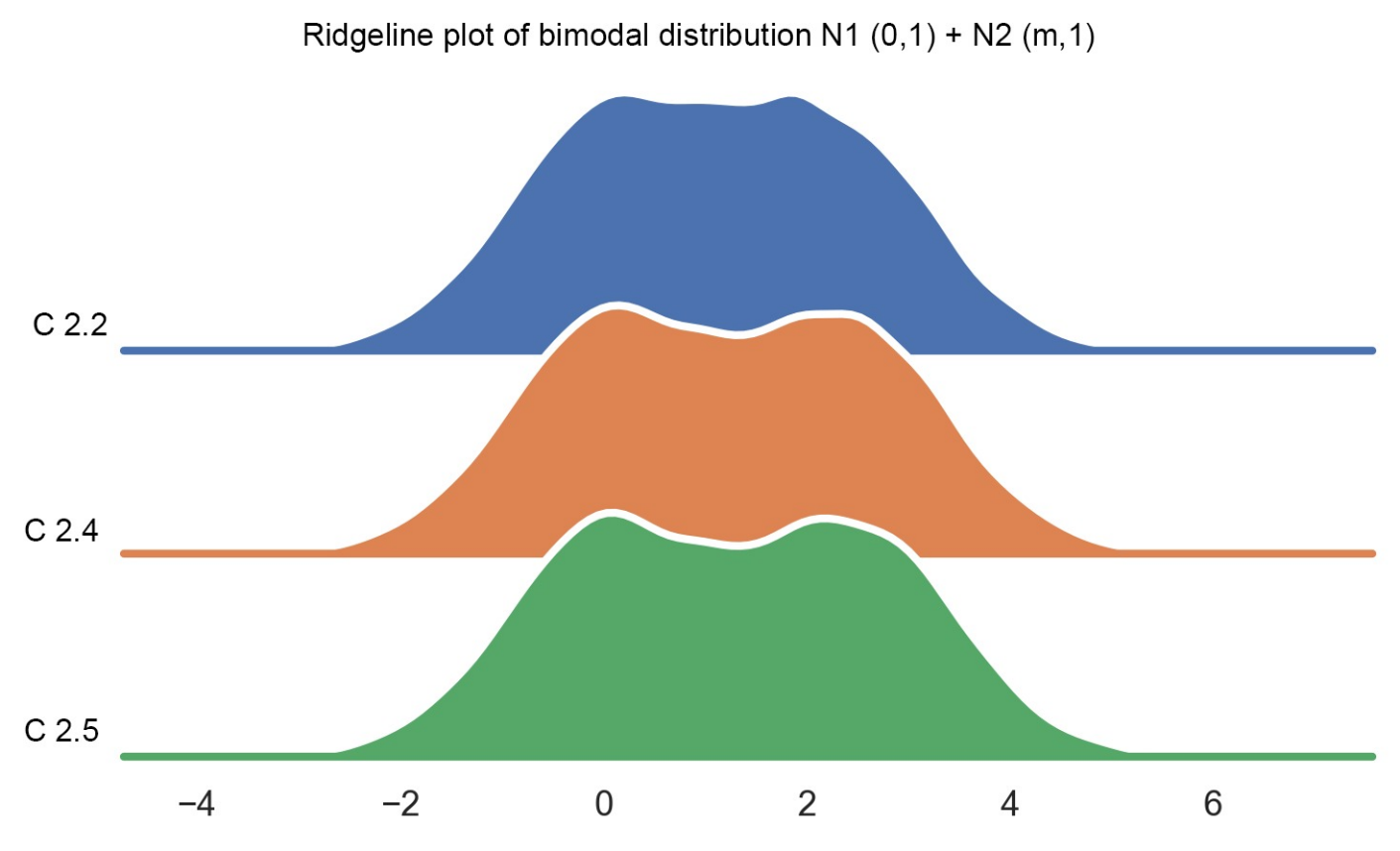


Fig B: Data with bimodal distribution visualized as ridgeline plot in Python. Similar to the MD plot, the ridgeline plot shows the bimodality of these data.


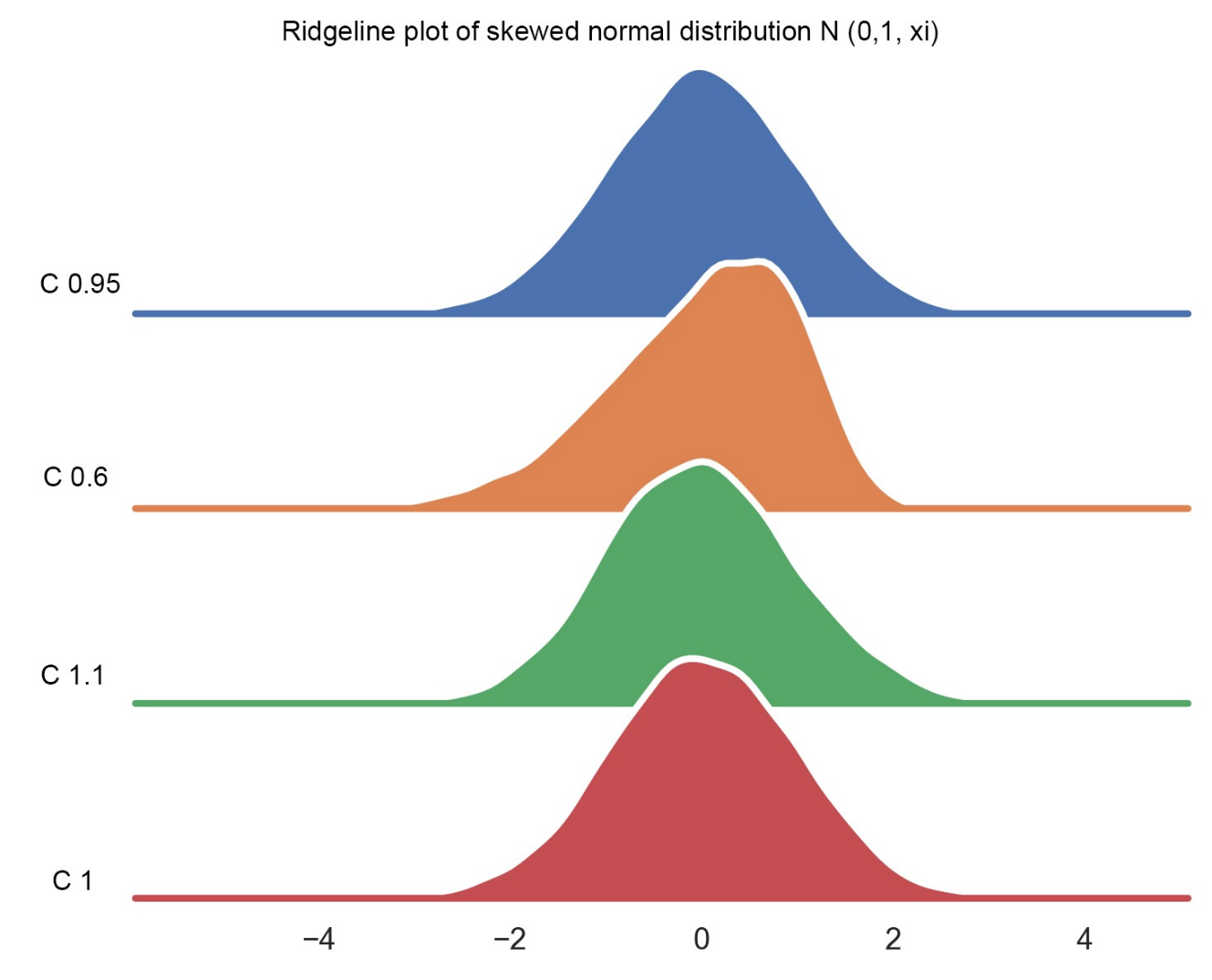


Fig C: The skewness of these unimodal distributions is visible in this ridgeline plot but slightly less sensitive than in the MD plot.


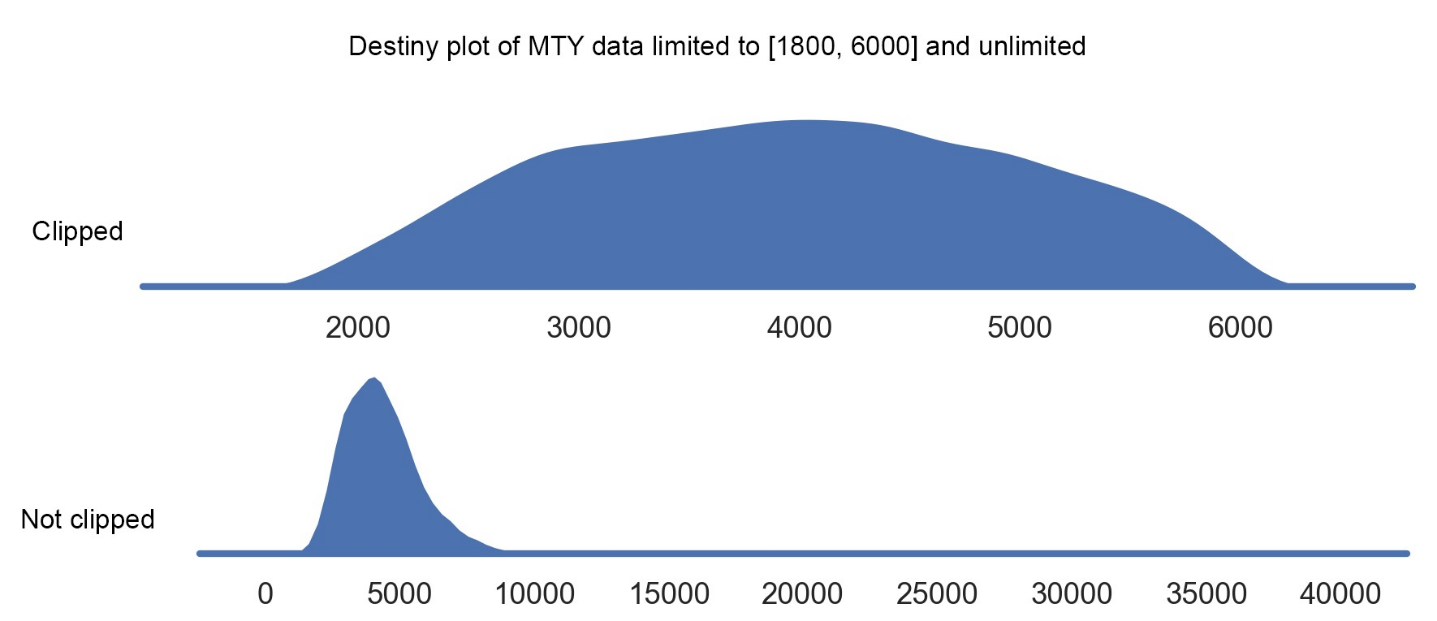


Fig D: The data for the upper visualization were limited to the range [1800, 6000]. Nevertheless, in contrast to the MD plot, the density plot goes beyond this range (especially beyond 6000).


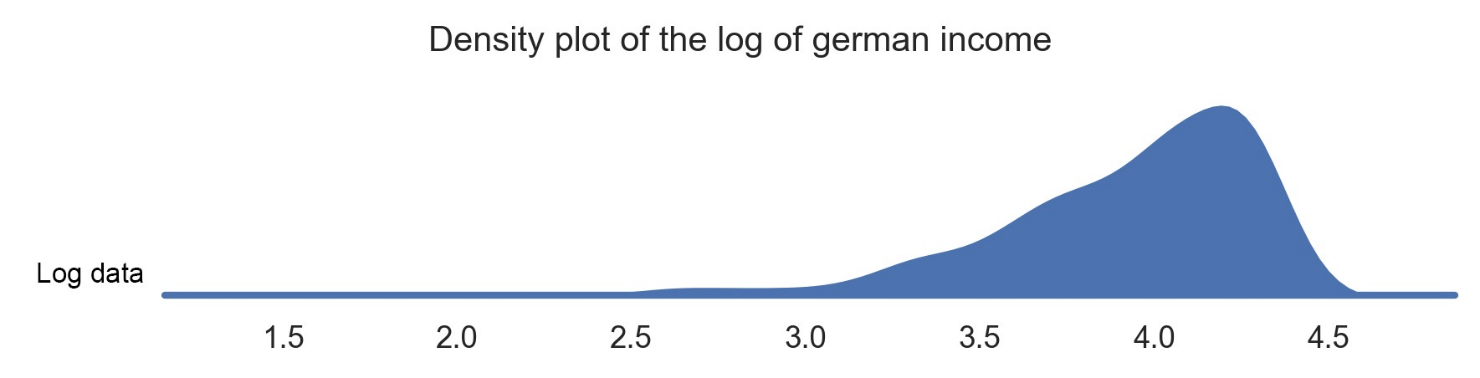


Fig E: Visualization of the log of German income. The density plot shows values above 4.35 and a less detailed, smoother distribution than the MD plot.


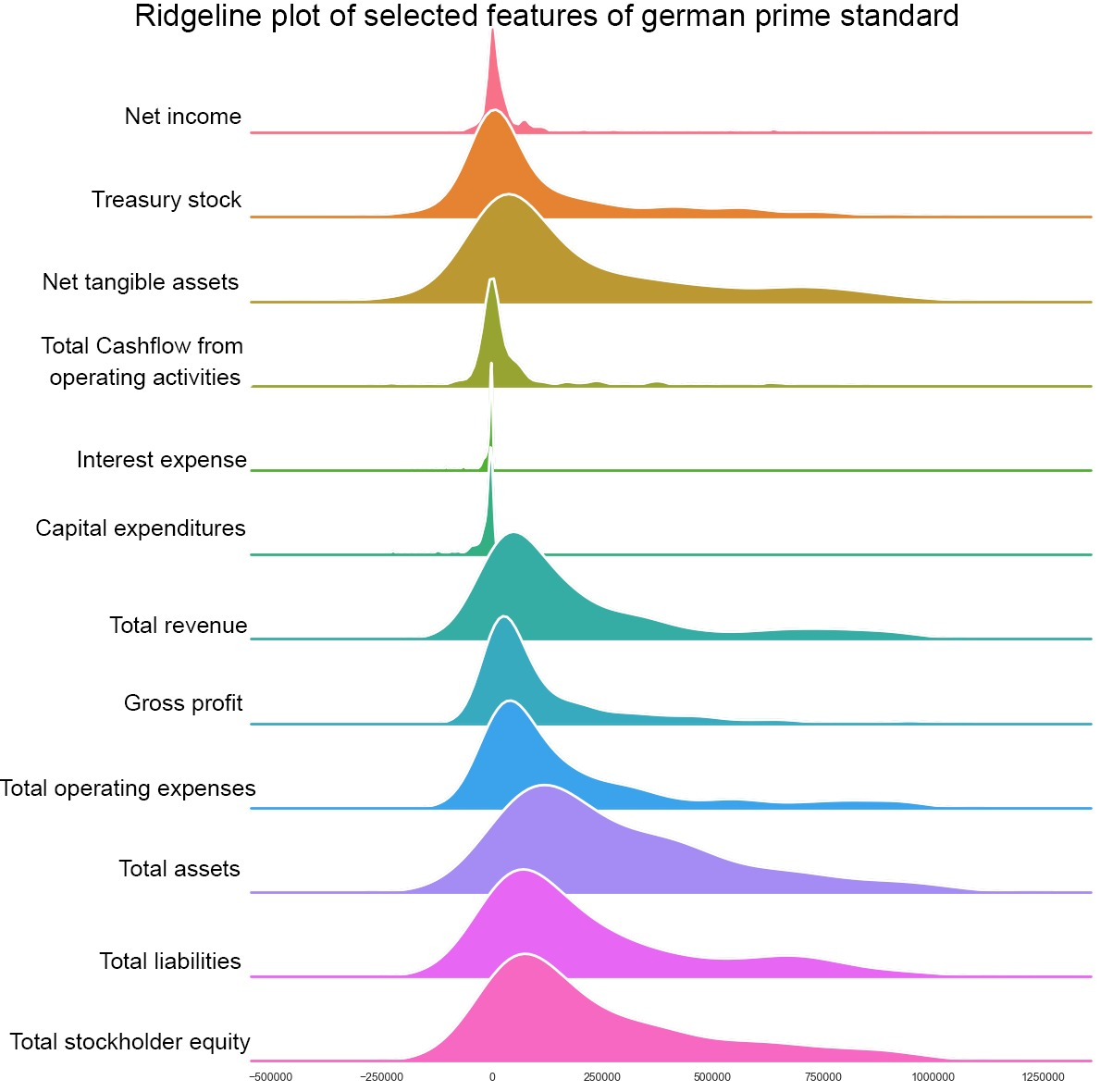


Fig F: Visualization of selected features from 269 companies on the German stock market reporting quarterly financial statements by the Prime standard. The ridgeline plot shows data above and below the limits [-250000, 1000000] and a less detailed, more smoothed distribution than the MD plot.


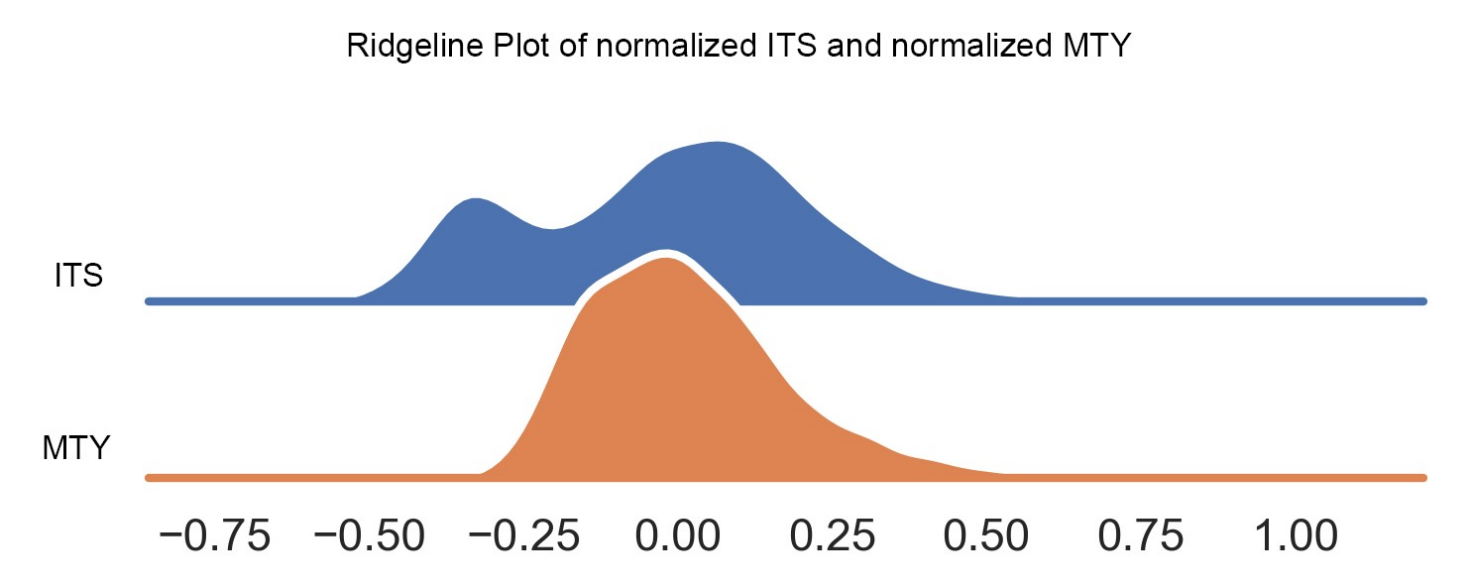


Fig G: Visualization of normalized data. The bimodality of the ITS is visible in the ridgeline plot and the MD plot.
