# Supplementary material for: Analyzing the fine structure of distributions
Source: PLoS One. 2020 Oct 14;15(10):e0238835. doi: 10.1371/journal.pone.0238835 (PMC7556505; doi:10.1371/journal.pone.0238835)

SI F: Violin Plot of ggplot2

1. Loading Data

The used datasets are either available in the DataVisualizations package or upon request via e-mail. The specific IO package used is accessible via GitHub., The datasets are loaded in the following Rmarkdown script. It should be noted that the ggplot2 package accesses data in the "long-table" format and the MD plot in the "wide-table" format. Therefore, the format is adjusted accordingly. Further, the high-dimensional dataset of stocks is divided into two parts in order to improve the visualization in this Rmarkdown script.

```
setwd(paste0(path, "/09Originale"))
DF_uniform = read.csv(
  "UniformSample.csv",
  stringsAsFactors = F,
  header = T,
  dec = "."
)
DF_bimodal = read.csv(
  "BimodalArtificial.csv",
  stringsAsFactors = F,
  header = T,
  dec = "."
)
DF_bimodal_longformat = reshape2::melt(DF_bimodal[, 2:4]) #no key
DF_skewed = read.csv(
  "SkewedDistribution.csv",
  stringsAsFactors = F,
  header = T,
  dec = "."
)
DF_skewed_longformat = reshape2::melt(DF_skewed[, 2:5]) #no key
DF_mun = read.csv(
  "MuncipalIncomeTaxYield_IncomeTaxShare.csv",
  stringsAsFactors = F,
  header = T,
  dec = "."
)

#simulate clipping
val1 = 1800
val2 = 6000
DF_mun$MTY_clipped = DF_mun$MTY
DF_mun$MTY_clipped[DF_mun$MTY < val1 | DF_mun$MTY > val2] = NaN

setwd(paste0(path, "/09Originale"))
##installing package via
#devtools::install_github("aultsch/DataIO",dependencies = T)
requireNamespace("dbt.DataIO")
LogIncomeV = dbt.DataIO::ReadLRN('SampleLogInome')
```

```
LogIncome = as.data.frame(LogIncomeV$Data)

stocksV = dbt.DataIO::ReadLRN('StocksData2018Q1')
StocksQ1 = as.data.frame(stocksV$Data)
Header = stocksV$Header
targets = c(
  'NetIncome_y',
  'TreasuryStock',
  'NetTangibleAssets',
  'ChangesInOtherOperatingActivities',
  'InterestExpense',
  'CapitalExpenditures',
  'TotalRevenue',
  'GrossProfit',
  'TotalOperatingExpenses',
  'TotalAssets',
  'TotalLiabilities',
  'TotalStockholderEquity'
)
ind = match(table = Header, targets)
#Splitting Features
DF_stocks_longformat_part1 = reshape2::melt(StocksQ1[, ind[1:6]])#select features
DF_stocks_longformat_part2 = reshape2::melt(StocksQ1[, ind[7:12]])#select features
```

2. Application of ggplot2::geom_violin

If default parameters are used, the geom_violin shows multimodality in the uniform distribution. In experiment I, bimodality is visible starting with m = 2.4 in the artificial data set. Bimodality is also visible in the natural data set of ITS. In experiment II, geom_violin performs better in the case of skewness than the violin plot of the package vioplot. Contrary to the bean plot, the data clipping is visible in experiment III.

```
requireNamespace("ggplot2")
ggplot2::ggplot(data = DF_uniform,
                mapping = ggplot2::aes_string(x = "Key", y = "UniformSample")) +

                ggplot2::geom_violin(scale = "width") + bw
```

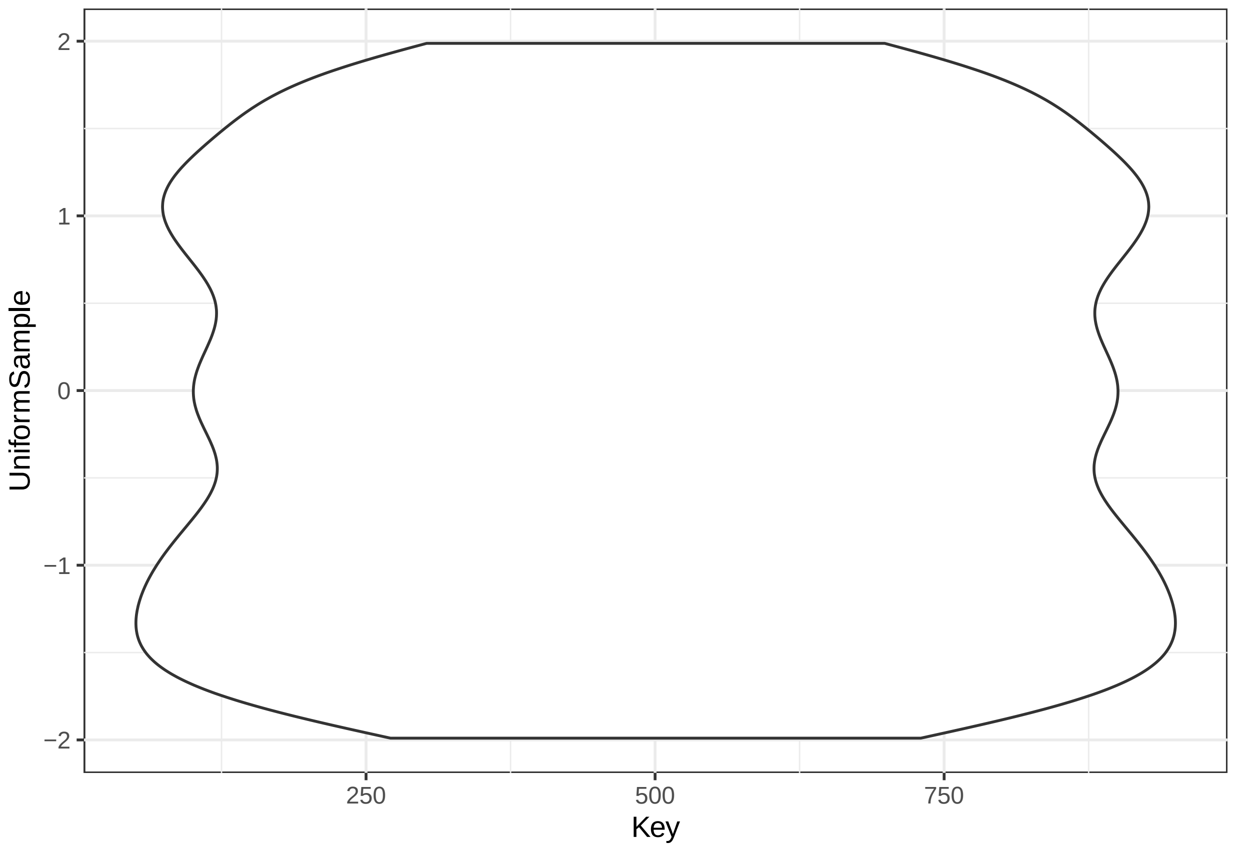


```
ggplot2::ggplot(data = DF_bimodal_longformat, ggplot2::aes_string(x = "variable", y =
    "value")) + ggplot2::geom_violin(scale = "width") + bw
```

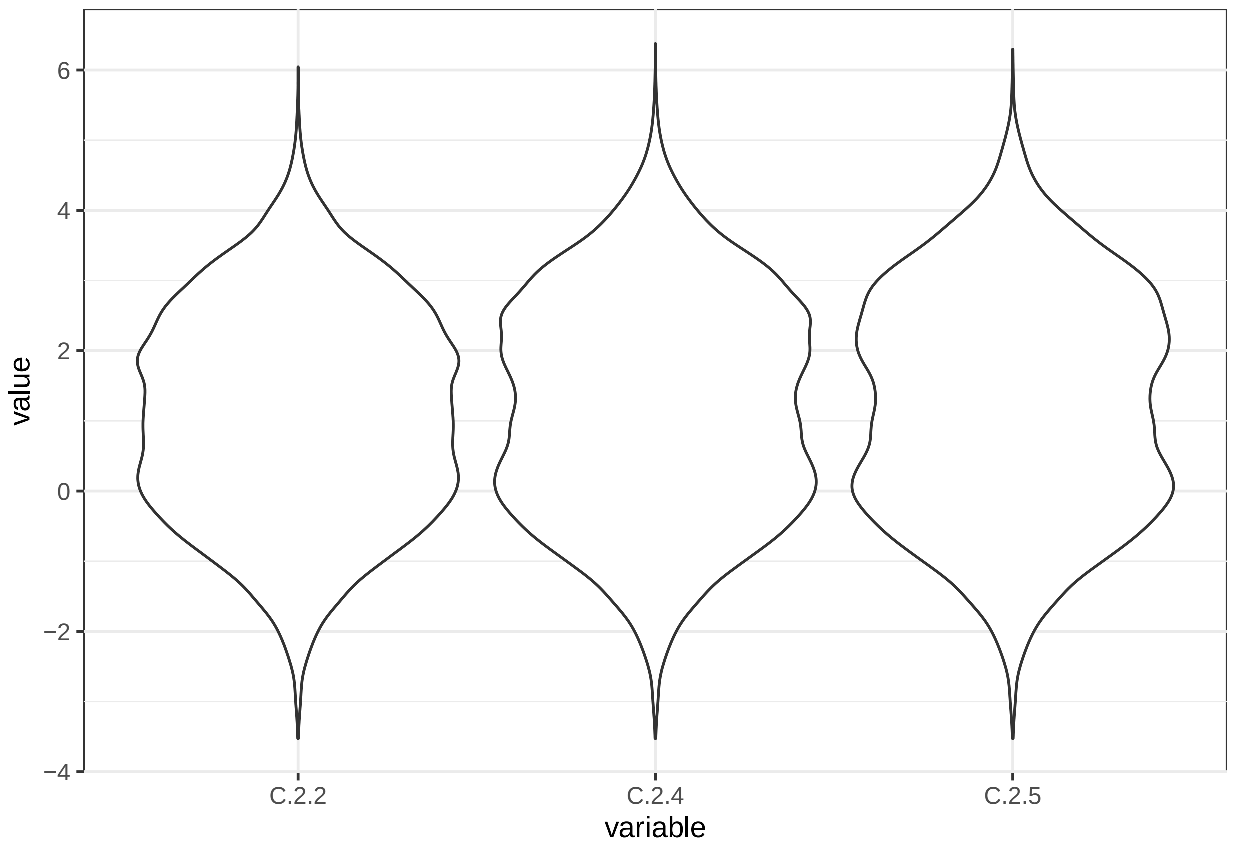


```
ggplot2::ggplot(data = DF_skewed_longformat, ggplot2::aes_string(x = "variable", y =
            "value")) + ggplot2::geom_violin(scale = "width") + bw
```

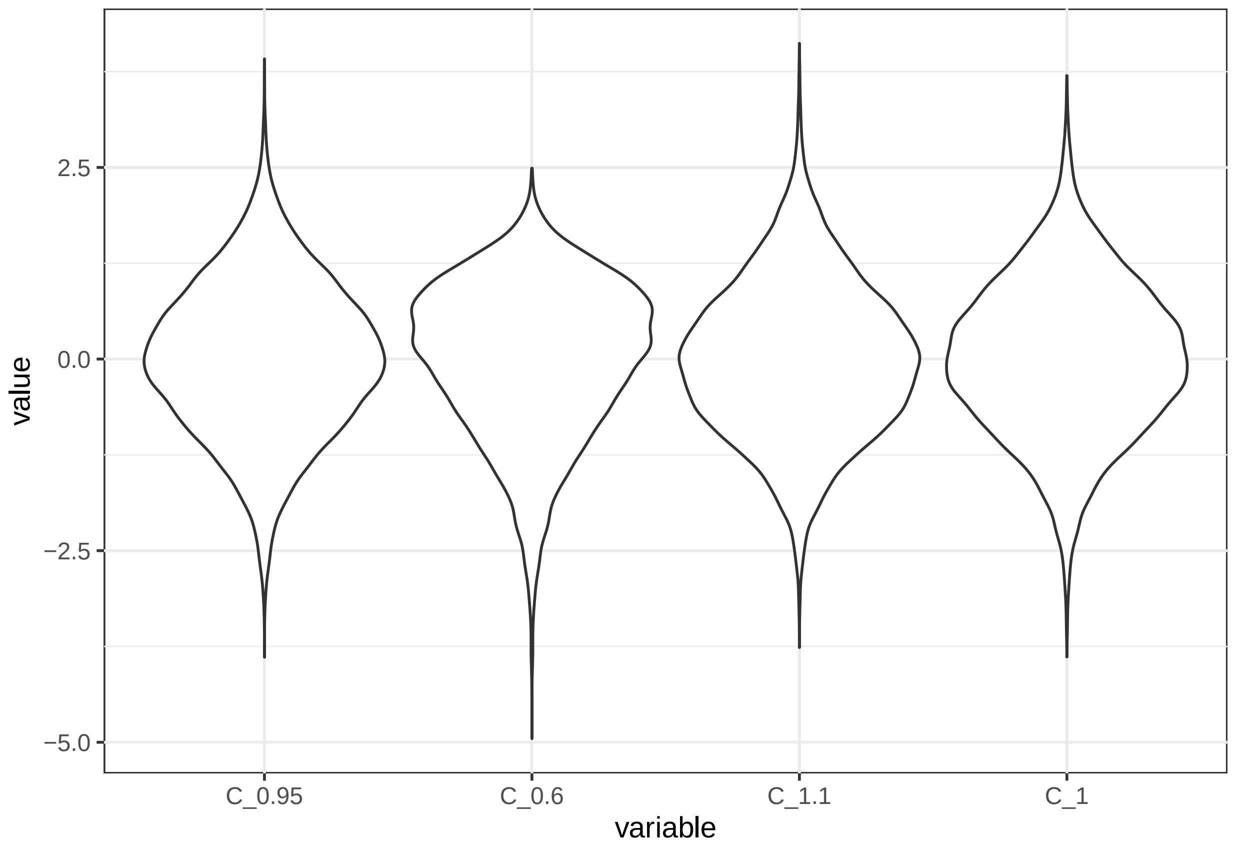


```r
ggplot2::ggplot(data = DF_mun,
                mapping = ggplot2::aes_string(x = "Key", y = "ITS")) +

                ggplot2::geom_violin(scale = "width") + bw
```

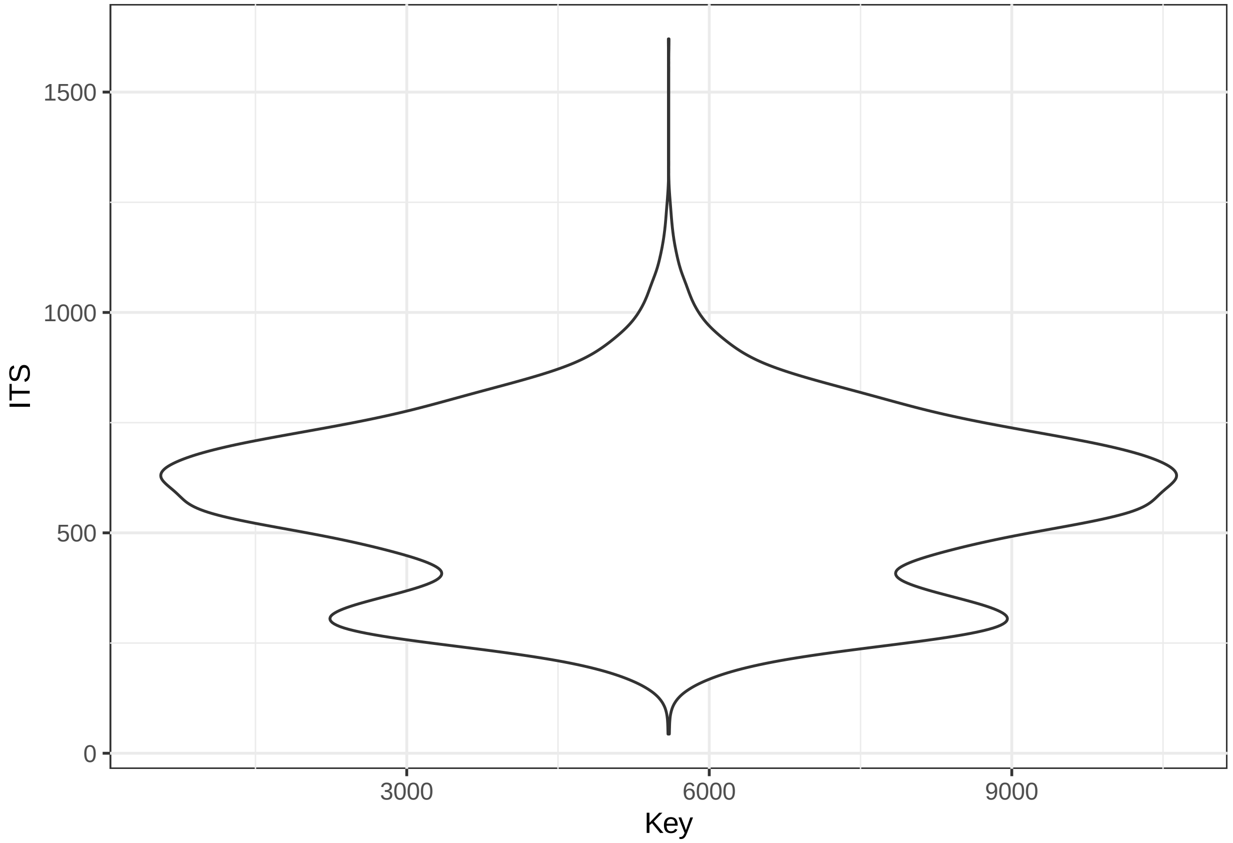


```r
ggplot2::ggplot(data = DF_mun,
                mapping = ggplot2::aes_string(x = "Key", y = "MTY_clipped")) +

                ggplot2::geom_violin(scale = "width") + bw
```

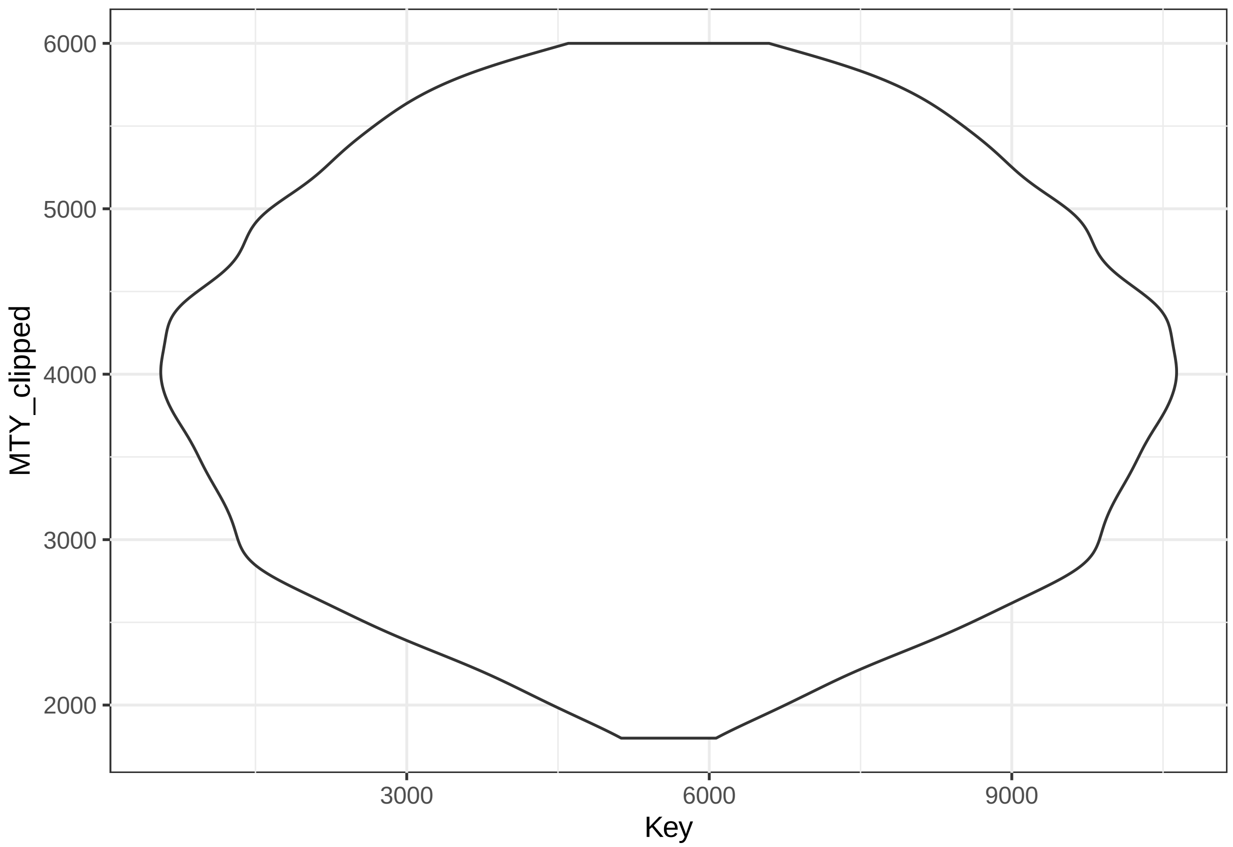

3. Plotting Natural Datasets with ggplot2::geom_violin

In experiment IV, using the data of log income, the geom_violin has a strong tendency to underestimate the density towards the maximum value if the default parameter setting is used. In experiment V, in the first part of the high-dimensional dataset, the kurtosis of the features "TreasuryStock" and "NetTangibleAssets" are overestimated. In the second part of the high-dimensional dataset, the skewness is underestimated, and multimodality is invisible.

```
requireNamespace("ggplot2")
library(ggExtra)
ggplot2::ggplot(data = LogIncome,
                mapping = ggplot2::aes_string(x = "Data_ind", y = "LogData")) +

                ggplot2::geom_violin(scale = "width") +

                ggplot2::xlab('Germans Log Income') + bw
```

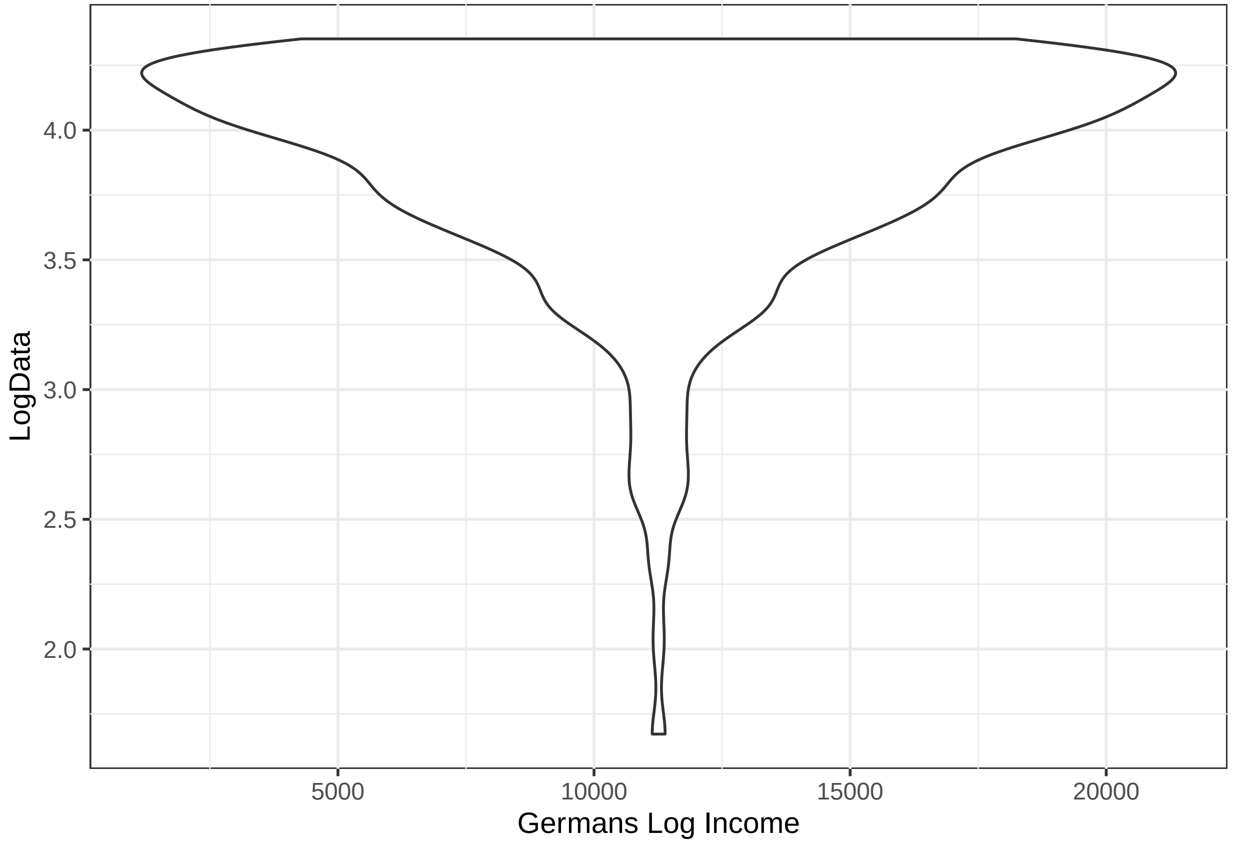


```
ggplot2::ggplot(data = DF_stocks_longformat_part1, ggplot2::aes_string(x =
         "variable", y = "value")) +ggplot2::geom_violin(scale = "width") +
          ggplot2::ylim(-200000, 1000000) + ggExtra::rotateTextX() + bw
```

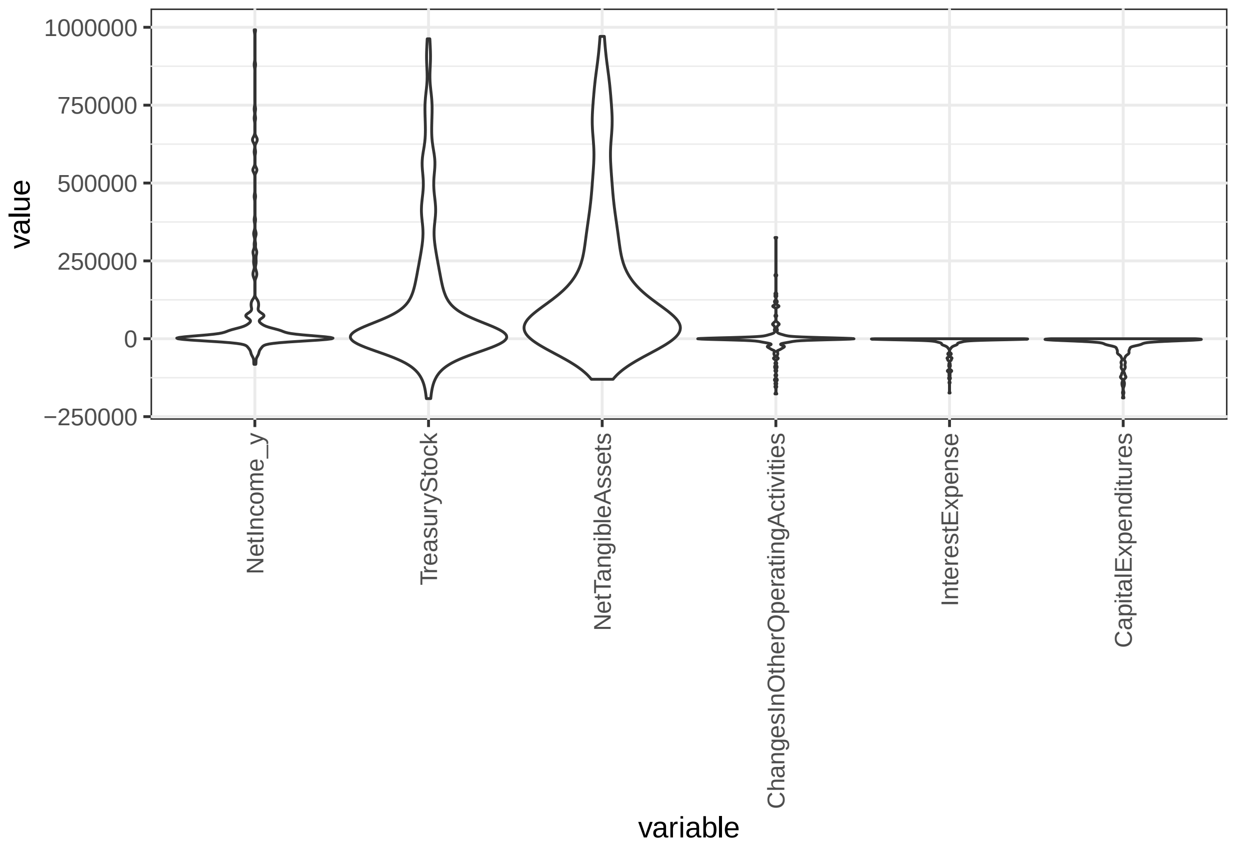


```
ggplot2::ggplot(data = DF_stocks_longformat_part2, ggplot2::aes_string(x =
        "variable", y = "value")) + ggplot2::geom_violin(scale = "width") +
        ggplot2::ylim(-200000, 1000000) + ggExtra::rotateTextX() + bw
```

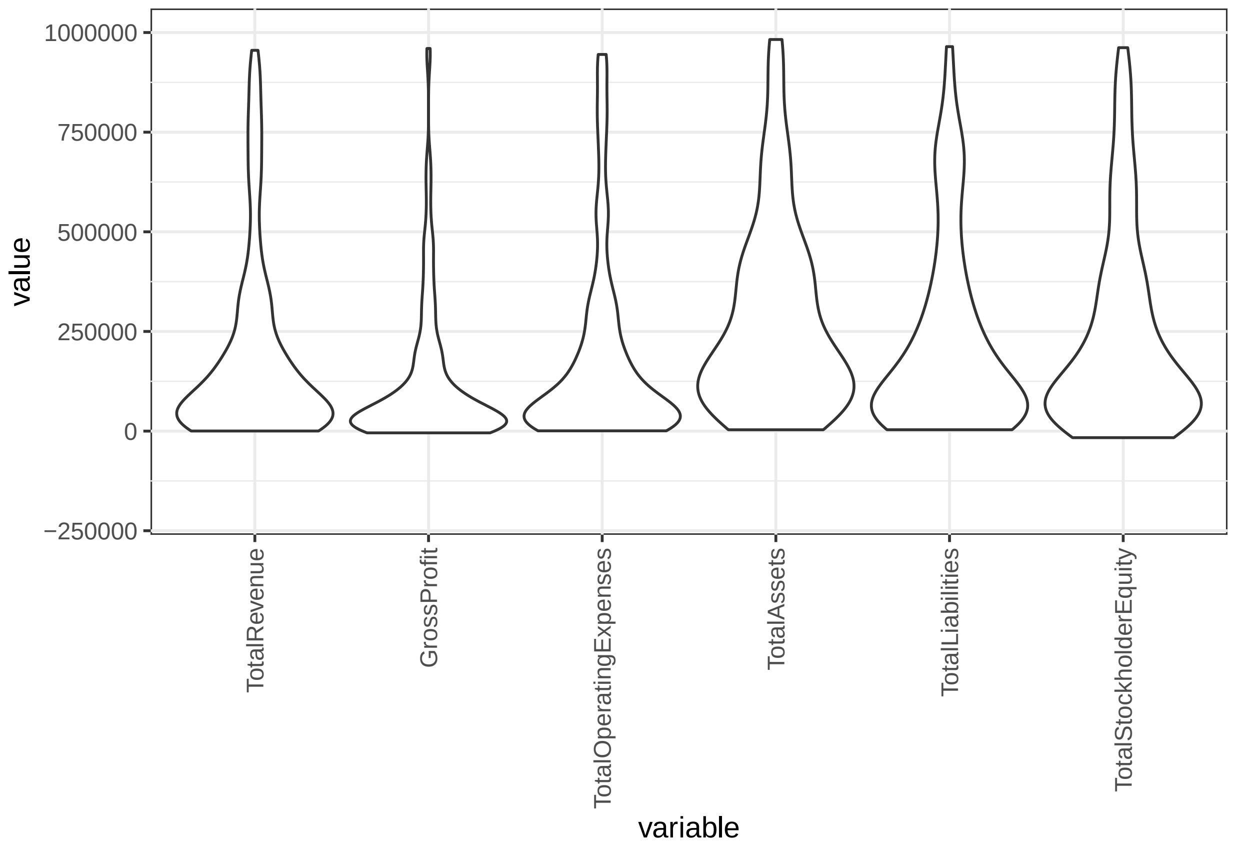


```r
library("DataVisualizations")
DataVisualizations::MDplot(as.matrix(StocksQ1[, ind[1:6]]), Ordering = "Columnwise") +
  ggplot2::ylim(-200000, 1000000) + bw
```

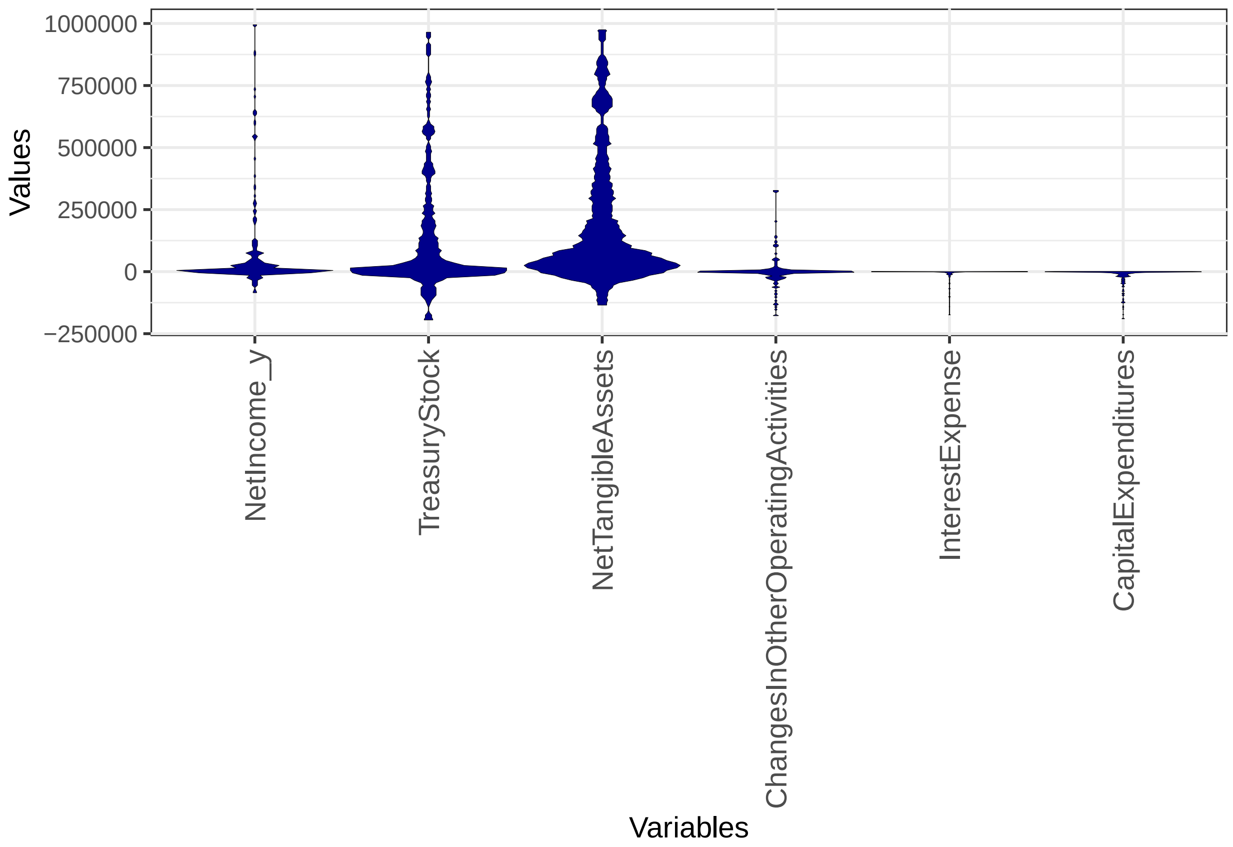


```r
DataVisualizations::MDplot(as.matrix(StocksQ1[, ind[7:12]]), Ordering = "Columnwise") +
  ggplot2::ylim(-200000, 1000000) + bw
```

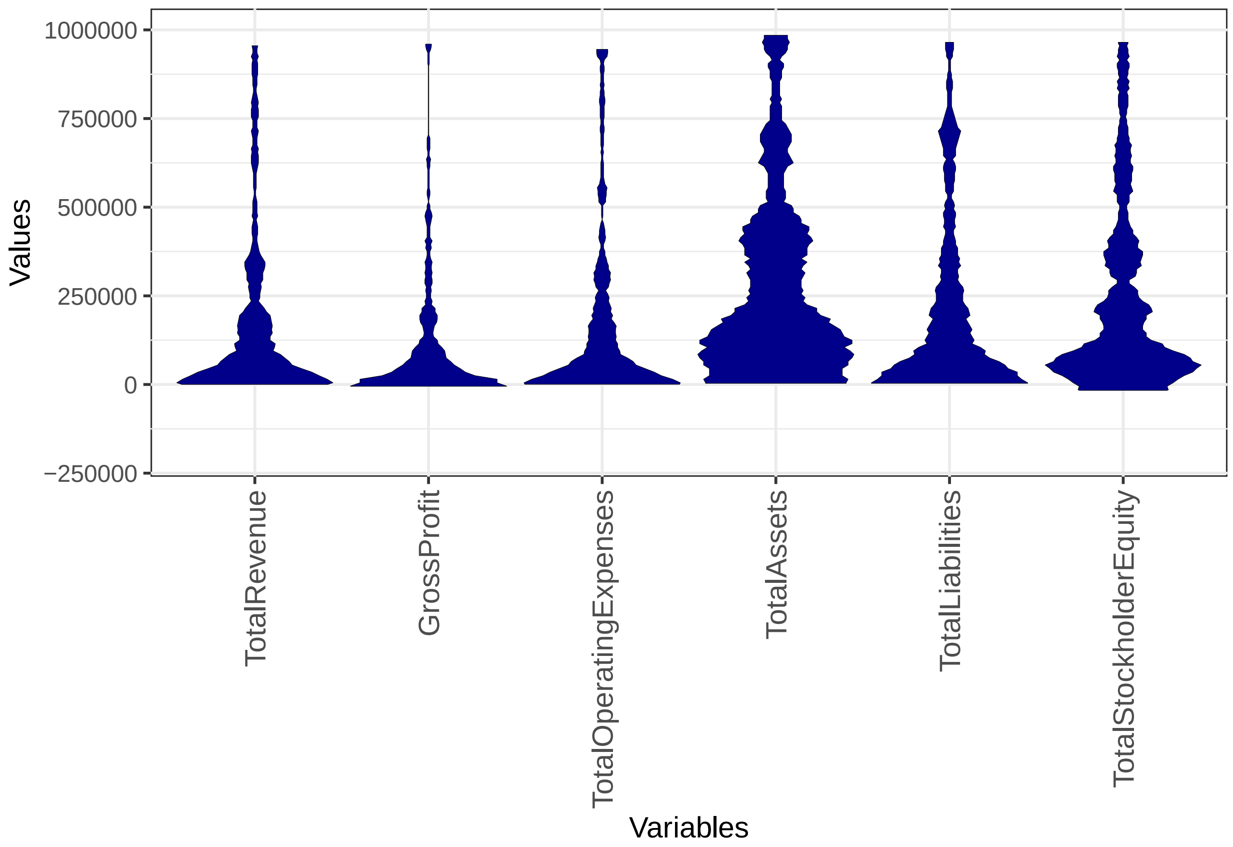

4. Setting of Parameters is Unfeasible

The argument can be made that every method can be adjusted with specific parameters so that the distribution is visualized correctly if prior knowledge is used. However, the same setting for one specific feature can result in an incorrect visualization of density for another feature. In the top figure, the violin plots of ggplot2 underestimate the density towards the maximum value and do not indicate multimodality. However, using this parameter setting, the visualization of the uniform distribution is improved considerably. In the bottom figure, the violin plots of ggplot2 shot the multimodality of log income correctly, and the estimation of density towards the maximum value is improved. However, the uniform distribution is incorrectly visualized as multimodal.

```
requireNamespace("ggplot2")
DataCombined = as.data.frame(cbind(
  LogIncome = LogIncome$LogData,
  Uniform = DF_uniform$UniformSample
))
DataCombined_long = reshape2::melt(DataCombined)

ggplot2::ggplot(data = DataCombined_long,
                mapping = ggplot2::aes_string(x = "variable", y = "value")) +

  ggplot2::geom_violin(kernel ="rectangular",adjust = 0.8,scale = "width") + bw
```

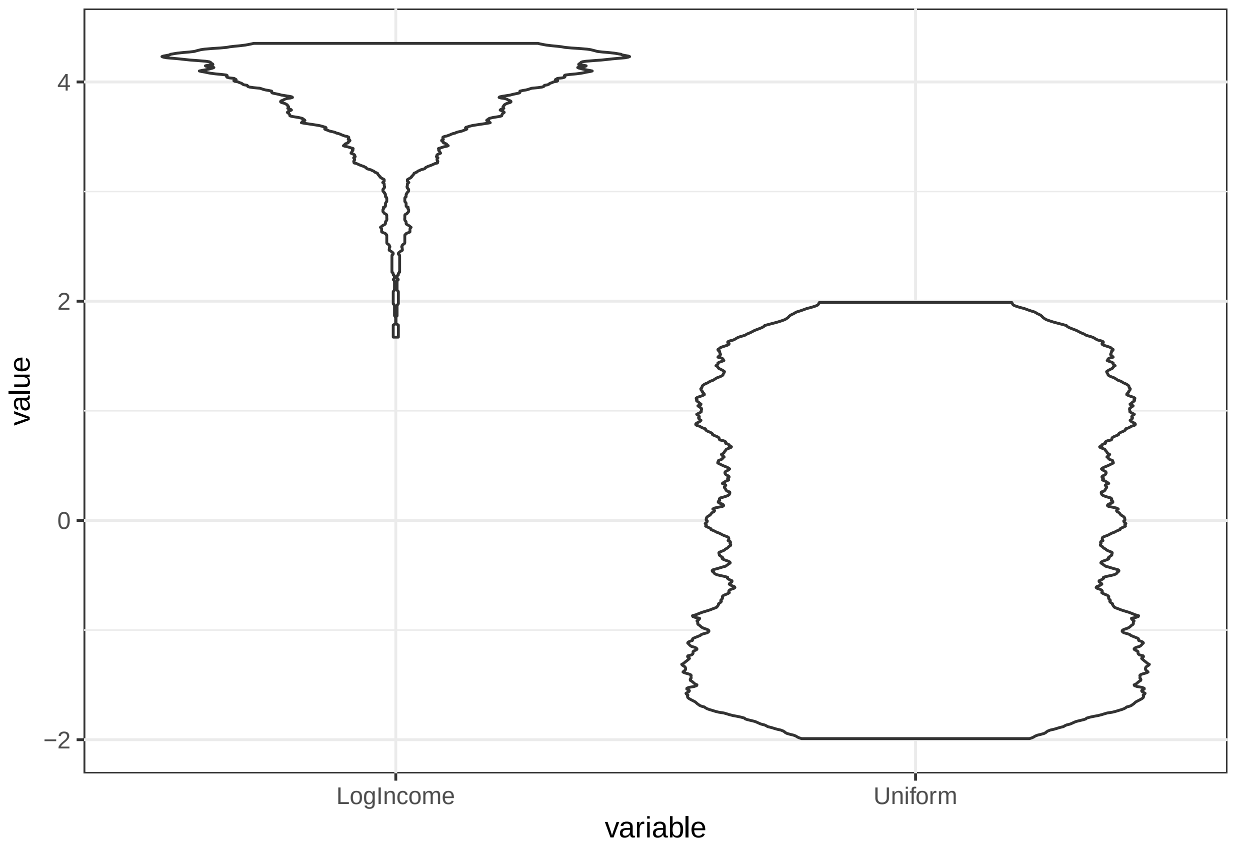


```
ggplot2::ggplot(data = DataCombined_long,
                mapping = ggplot2::aes_string(x = "variable", y = "value")) +

  ggplot2::geom_violin(kernel = "triangular",adjust = 0.45, scale = "width") + bw
```

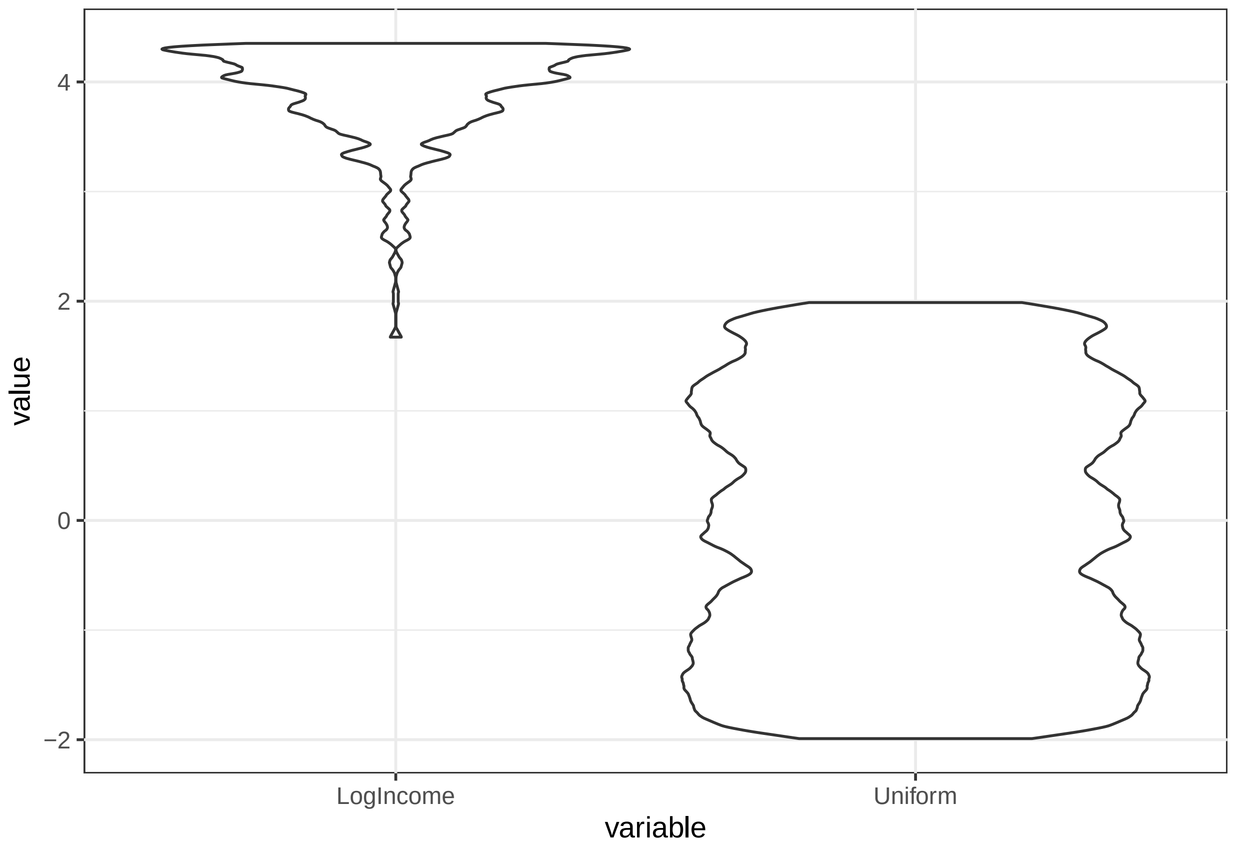


```r
library(DataVisualizations)
MDplot(as.matrix(DataCombined),
       Names = c("LogIncome", "UniformSample")) + bw
```

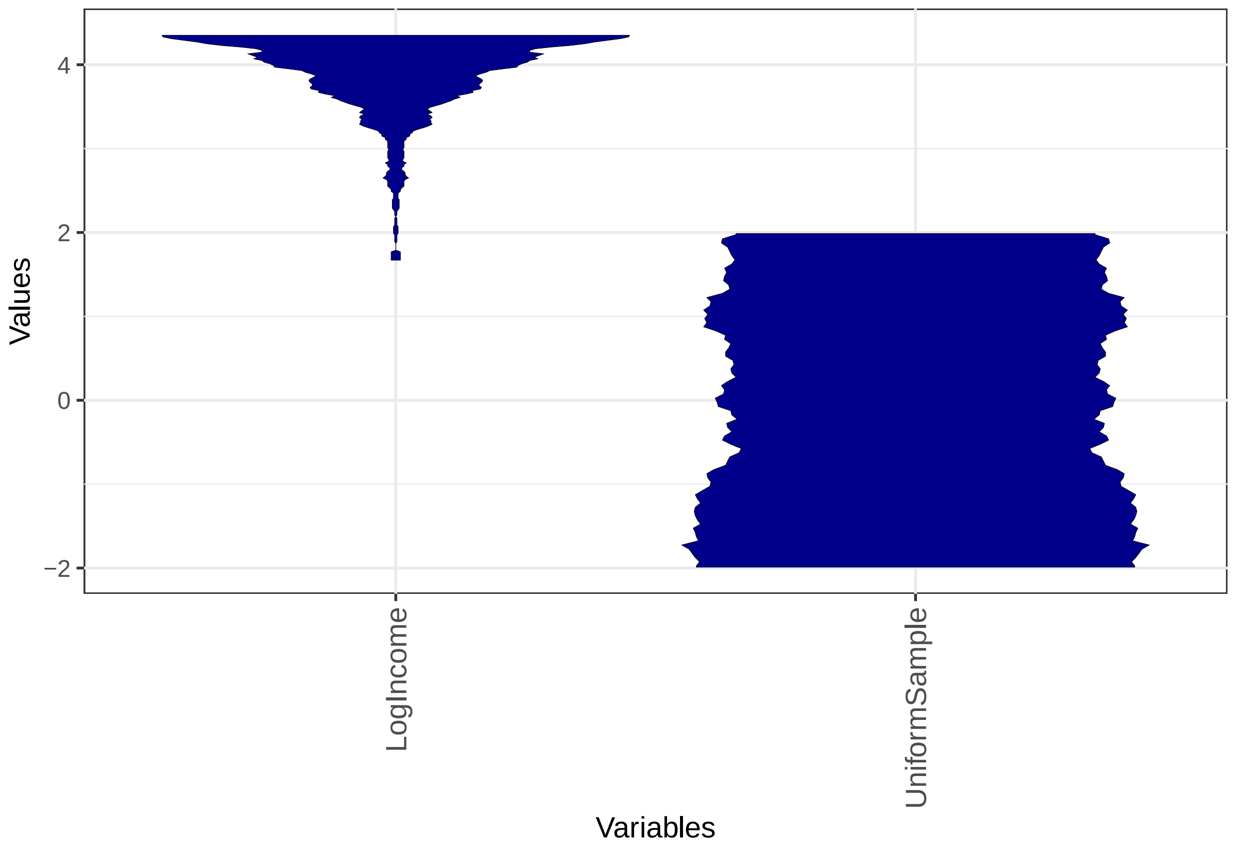

5. Discrete States

The error rates for the clustering methods that tried to reproduce the prior classification of the Lsun3D dataset taken from Thrun and Ultsch, 2020 are used here [57]. It should be noted that the k-means methods of KM and KM-ID12 differ in the initialization procedure. In the MD plot, it is clearly visible that KM and FKM have two quantized error states, contrary to PBC, ProClus and Orclus for which density has to be estimated. Other methods can only be described by a Dirac delta distribution visualized with a line. Contrary to the MD plot, it is evident that the violin plot of ggplot2 is unable to visualize discrete states.

The abbreviations are as follows: KM (k-means), KM-ID12 (specific Initialization procedure), RKM (Reduced k-means), FKM (Factorial k-means), PPC (Projection Pursuit Clustering) with either MD (MinimumDensity), MaximumClusterbility (MC) or NormalisedCut (NC).

```
setwd(paste0(path, "/09Originale"))
##installing package via
#devtools::install_github("aultsch/DataIO",dependencies = T)
requireNamespace("dbt.DataIO")
benchV = dbt.DataIO::ReadLRN('Lsun3D_Benchmarking')
Benchmarking = as.data.frame(benchV$Data)
Benchmarking_long = reshape2::melt(Benchmarking)

MDplot(Benchmarking) + bw
```

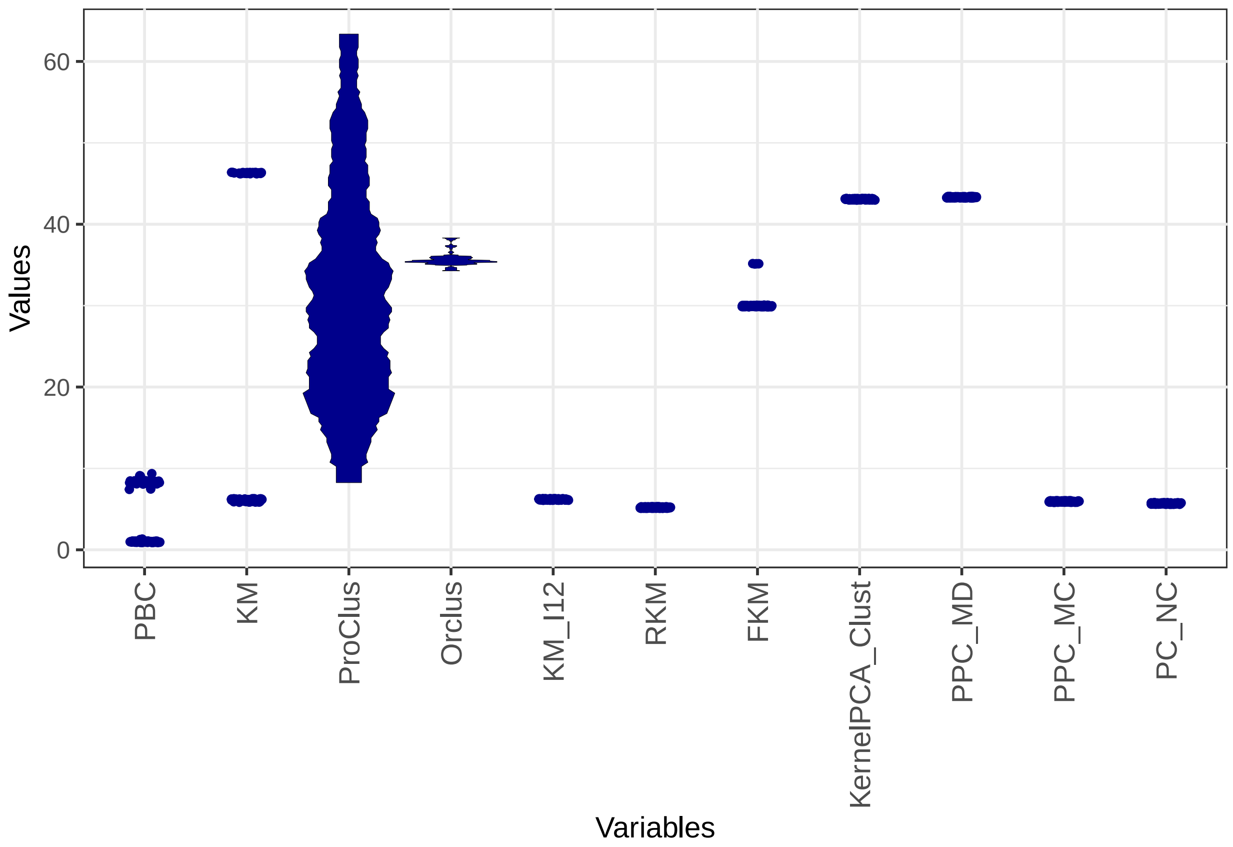


```r
requireNamespace("ggExtra")
ggplot2::ggplot(Benchmarking_long, ggplot2::aes_string(x = "variable", y = "value")) +
      ggplot2::geom_violin(scale = "width") + ggExtra::rotateTextX() + bw
```

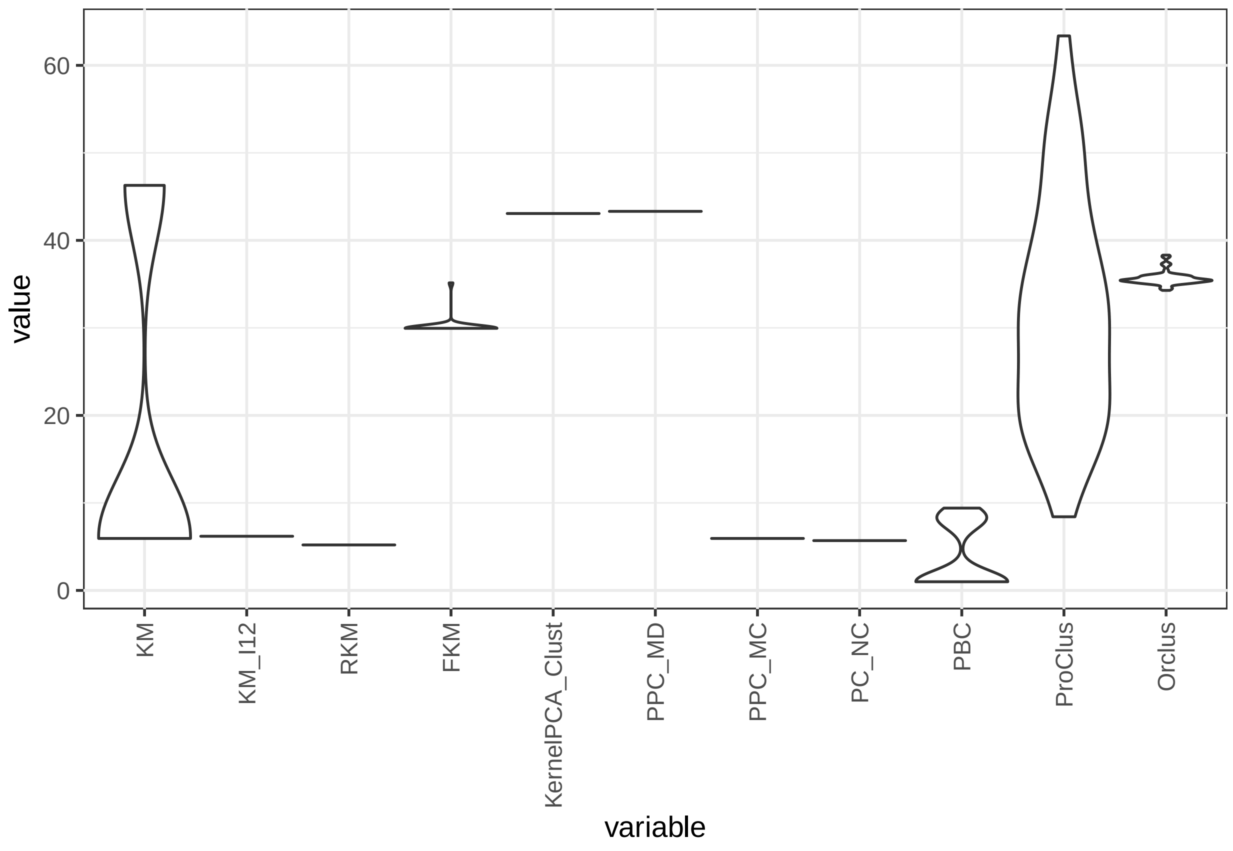

6. Multimodality With Gaps

Two features are taken from article of Hoffmann et al., 2020 in this example [59]. The two features are bimodal and have gaps in the value range that are marked with two red lines. The violin plot of ggplot2 visualizes the density between the two modes of the features, whereas the MD plot does not visualize any existing density in the gaps.

```
setwd(paste0(path, "/09Originale"))
##installing package via
#devtools::install_github("aultsch/DataIO",dependencies = T)
requireNamespace("dbt.DataIO")
Bimodal2V = dbt.DataIO::ReadLRN('MultimodalityWithGap')
HeaderBimodal = Bimodal2V$Header
Bimodal2 = as.data.frame(Bimodal2V$Data)
Bimodal2_long = reshape2::melt(Bimodal2)

#Distribution Analysis

#geom_violin
requireNamespace("ggplot2")
ggplot2::ggplot(data = Bimodal2_long, ggplot2::aes_string(x = "variable", y =

           "value")) + ggplot2::geom_violin(scale = "width") +

              ggplot2::geom_hline(yintercept = 0.9825, col ="red") +
```

```
                ggplot2::geom_hline(yintercept = 0.995, col = "red") + bw
```

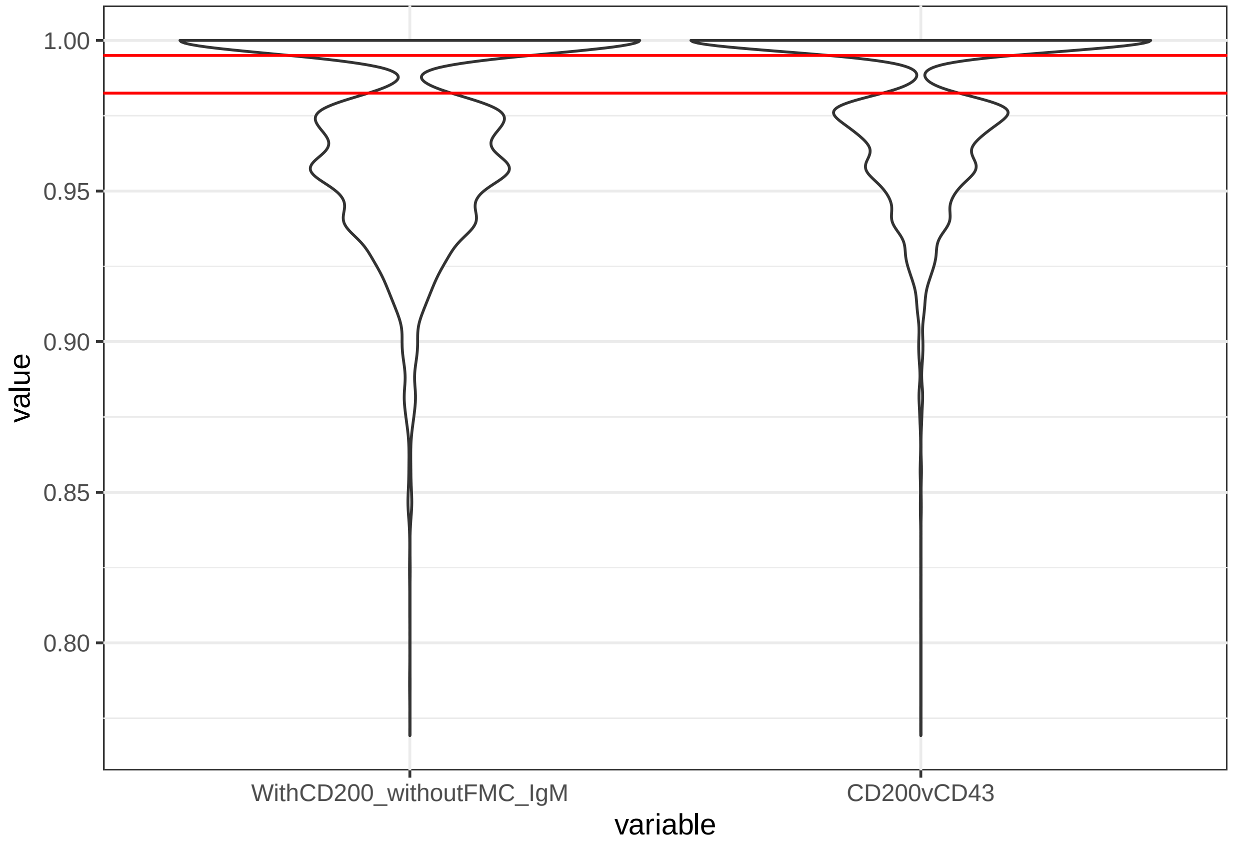


```
#MDplot
library("DataVisualizations")
DataVisualizations::MDplot(as.matrix(Bimodal2)) + ggplot2::geom_hline(yintercept = 0.9825,

                          col ="red") + ggplot2::geom_hline(yintercept = 0.995, col = "red") +
                          bw
```

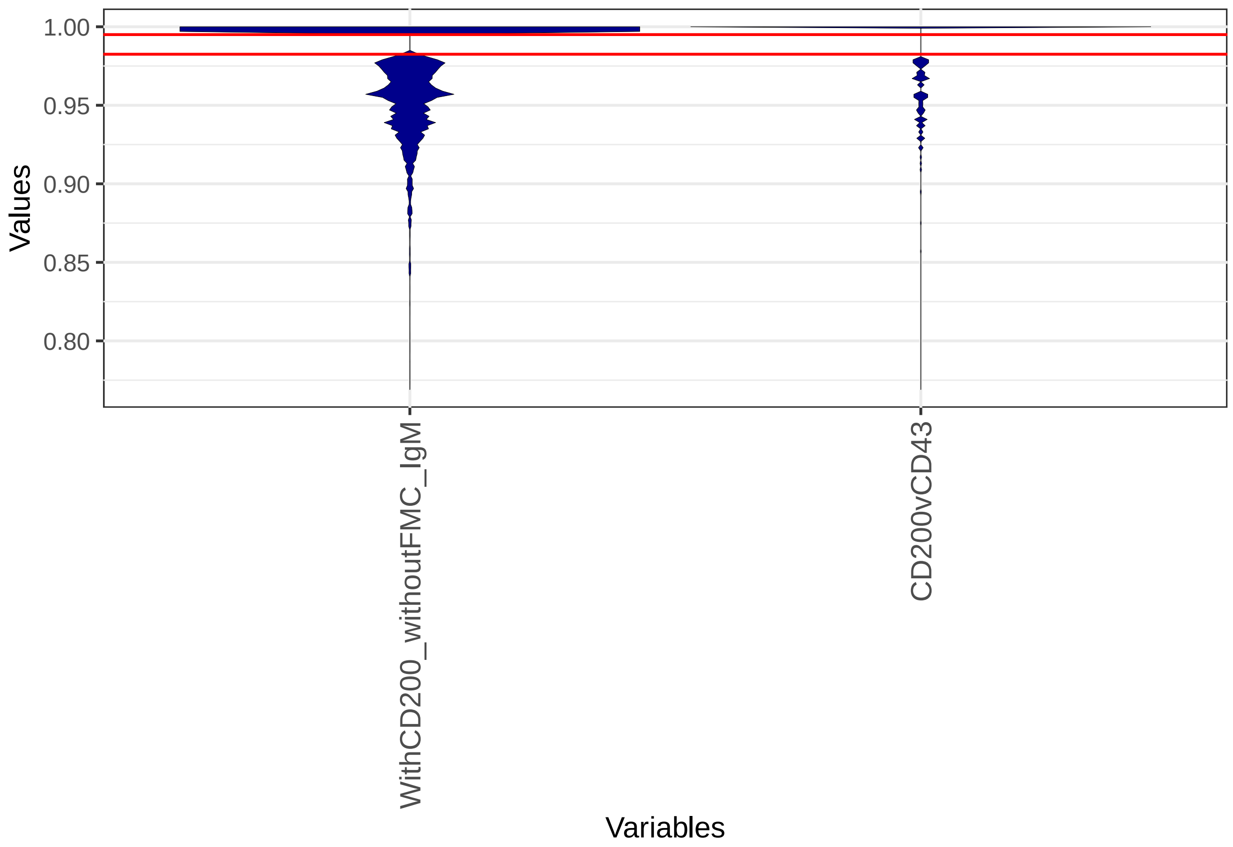

7. Distribution Analysis

Distribution analysis of the dataset is performed, using various methods, in order to more substantiate the abovementioned argument that MD plot visualizes the density correctly wheareas geom_violin does not. As a result, the QQ plot and histogram agree with the density estimation, meaning that apparent gaps exist in the data. Hence, the MD plot visualizes this data correctly, and the geom_violin plot does not.

```
requireNamespace("DataVisualizations")
i = 1
DataVisualizations::InspectVariable(Bimodal2[, i], HeaderBimodal[i])
```

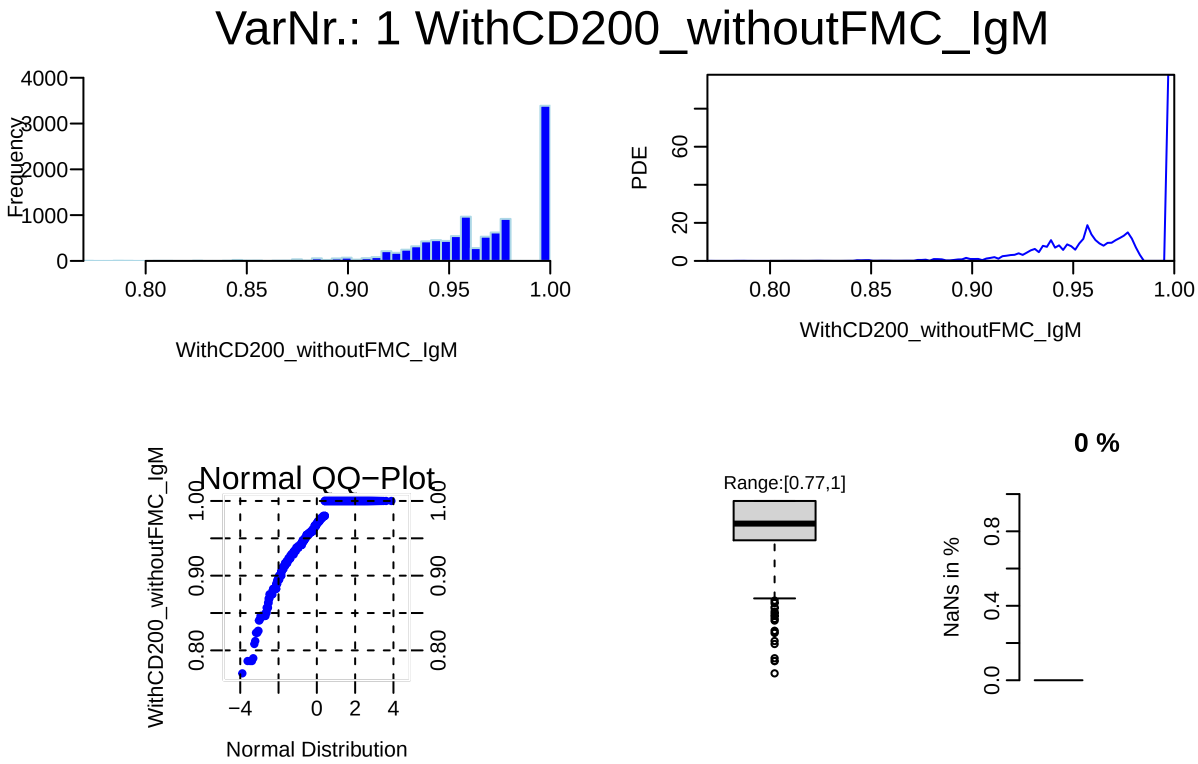


```
i = 2
DataVisualizations::InspectVariable(Bimodal2[, i], HeaderBimodal[i])
```

VarNr.: 1 CD200vCD43

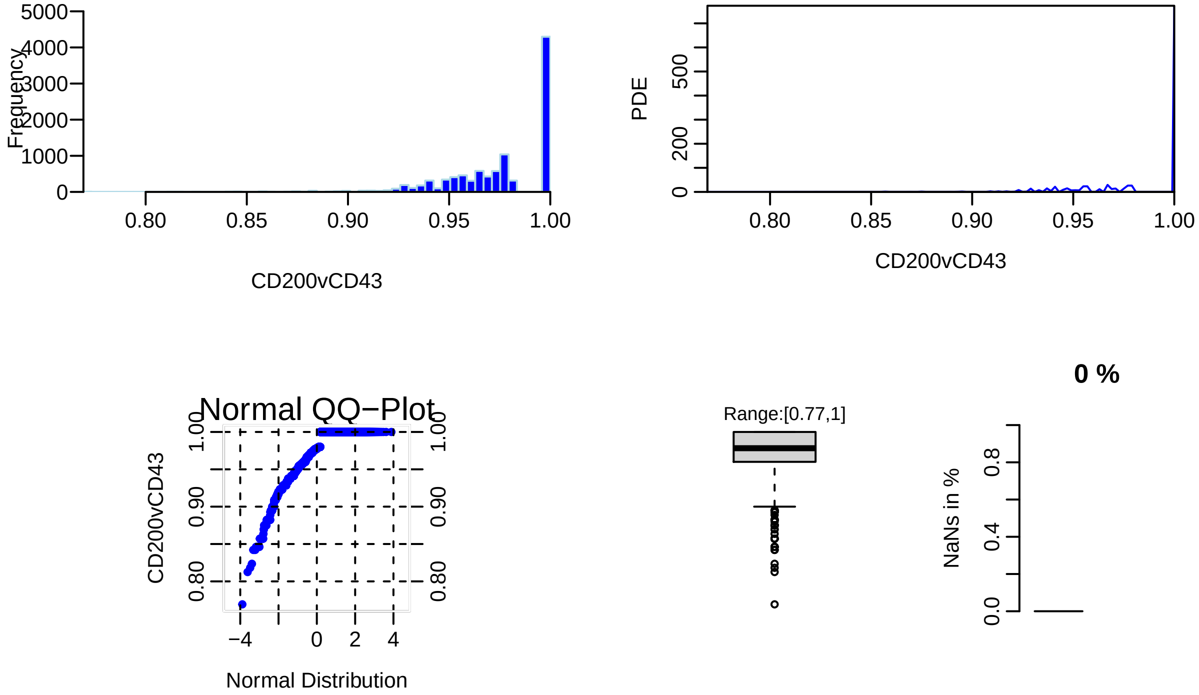

Supplement: S6 File — (PDF) [file pone.0238835.s006.pdf]
