# Supplementary material for: Analyzing the fine structure of distributions
Source: PLoS One. 2020 Oct 14;15(10):e0238835. doi: 10.1371/journal.pone.0238835 (PMC7556505; doi:10.1371/journal.pone.0238835)
Supplement: S7 File — (DOCX) [file pone.0238835.s007.docx]

md_plot: A Python Package for Analyzing the Fine Structure of Distributions

# Introduction

In data analysis of industrial topics such as the quality assurance of production facilities or the analysis of customer behavior, we constantly encounter univariate distributions of all kinds. From unimodal Gaussians to symmetric or skewed distributions to multimodal distributions It is of immense importance to determine the exact shape of the distribution in order to be able to select the correct further analysis steps and to be able to draw correct conclusions. Of special interest is the question, whether the given empirical distribution is composed of two or more distinct subsets of data points. Such subsets give hints to the existence of different states of the data producing process, such as, for example, healthy vs. sick patients or the existence of different diseases or treatments.

Conventional visualization methods of univariate probability density distributions have problems in the distinction of uniform versus multimodal distributions and in visualizing capped skewed distributions correctly. With the mirrored density plot, a visualization method more suitable for these applications was postulated [Thrun/Ultsch, 2019] in the programming language R, which is now extended to python.

The Python package *md_plot* is an implementation of the MD-Plot function of the R package *DataVisualizations* on CRAN [Thrun et al., 2018]. The use of the Python package is described in this technical report. The usage of the R package is described in the Vignette on CRAN.

# Basic Usage

## 2.1 Inbuild Samples

The md_plot package offers several samples to make getting started using the MD-Plot easier. To do this, call the function load_examples, which returns a dictionary. This dictionary contains several keys, which each provide access to a single pandas dataframe.

- *from md_plot import load_examples*
- *dctExamples = load_examples()*


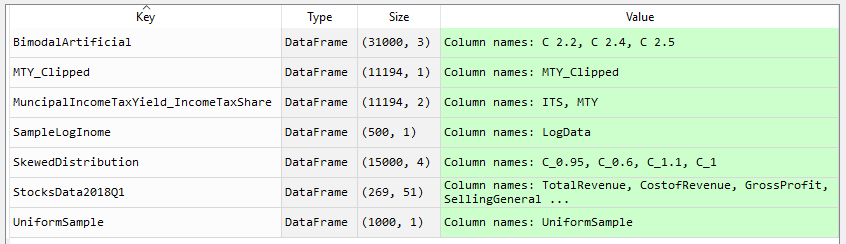


**Fig. 1:** Return of the load_examples function. The sample data is contained in a dictionary as pandas dataframes and can be used in the MDplot to reproduce the visualizations.

These dataframes can now be used in the md_plot function.

## 2.2 Visualization

The MDplot function of the md_plot package accepts pandas series (vectors) and pandas dataframes (matrices) or anything that converts simply into these two data structures (for example, lists and numpy arrays) as input. Preprocessing depending on the analysis can be before done before visualization. For example the capping of values:

- *from md_plot import MDplot*
- *dfMTY = dctExamples["MTY_Clipped"]*
- *dfMTY = dfMTY[(dfMTY["MTY_Clipped"] >= 1800) & (dfMTY["MTY_Clipped"] <= 6000)]*
- *MDplot(dfMTY)*


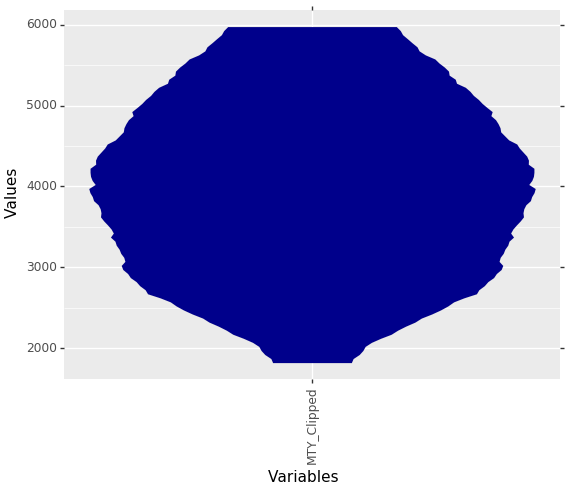


**Fig. 2:** MD-Plot of capped municipality income tax yield (MTY) of Germany municipalities of 2015. Good to see are the clear capping limits at which the were cropped for the visualization.

The function returns a ggplot object, but you can get additional information by setting the *OnlyPlotOutput* parameter to False.

- *dctResult = MDplot(dctExamples['BimodalArtificial'], OnlyPlotOutput=False)*

**2.3 Changing Layout**

The layout of the ggplot object returned by the MDplot can be modified by adding additional ggplot objects, e.g. a title.

- *import plotnine as p9*
- *MDplot(dfMTY) + p9.labels.ggtitle('Capped MTY data') + p9.labels.ylab('PDE') + p9.labels.xlab('Variables') + p9.theme_seaborn()*
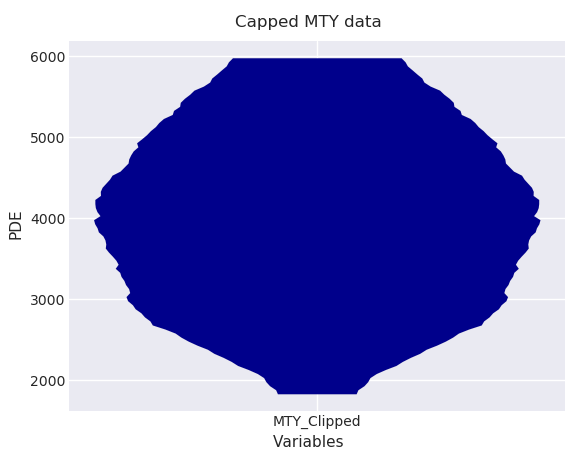


**Fig. 3:** MD plot of capped MTY data with title, changed axis labels and in seaborn theme.

Further information on the layout design of ggplots in plotnine can be found in the official documentation at https://plotnine.readthedocs.io.

# Advanced Usage

## 3.1 Draw a Gaussian distribution

The parameter *RobustGaussian* is used to activate or deactivate an overlay of a Gaussian distribution (this activated by default). The Gaussian distribution will only be drawn if several statistical tests have shown that the data is unimodal and not skewed. For changing the visual appearance of the Gaussian distribution, the parameters *GaussianColor* and *GaussianLwd* (line width) are provided.

- *MDplot(dctExamples['BimodalArtificial'], RobustGaussian=False)*


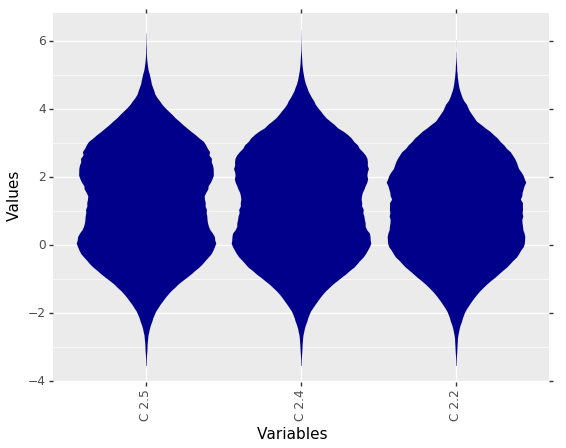


**Fig. 4:** Visualization of bimodal data without drawn Gaussian distribution.

- *MDplot(dctExamples['BimodalArtificial'], GaussianColor='green', GaussianLwd=2.5)*
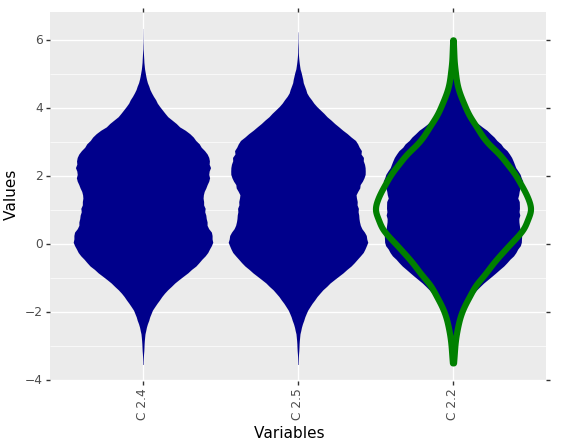


**Fig. 5:** Visualization of bimodal data with a drawn Gaussian distribution.

## 3.1 Draw a Box Plot

The parameters *BoxPlot* (deactivated by default) and *BoxColor* are used for plotting a boxplot over each MD-Plot.

- *MDplot(dctExamples['* *UniformSample'], BoxPlot = True)*
-
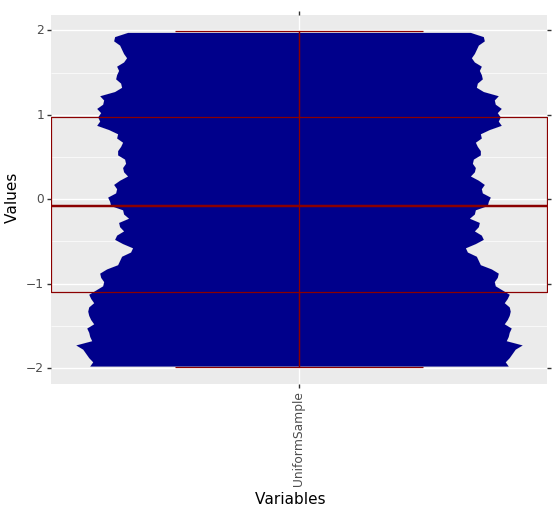


**Fig. 6:** Uniformly distributed data with drawn Box Plot. The Box Plot alone would suggest a Gaussian distribution.

## 3.2 Sampling

In order to avoid too long calculation durations, the MD-Plot determines a uniformly distributed, random sample. This is controlled by the parameter *SampleSize* (default: 500000 elements / cells).

- *dfMTY = dctExamples["MTY_Clipped"]*
- *dfMTY = dfMTY[(dfMTY["MTY_Clipped"] >= 1800) & (dfMTY["MTY_Clipped"] <= 6000)]*
- *MDplot(dfMTY, SampleSize=5000)*


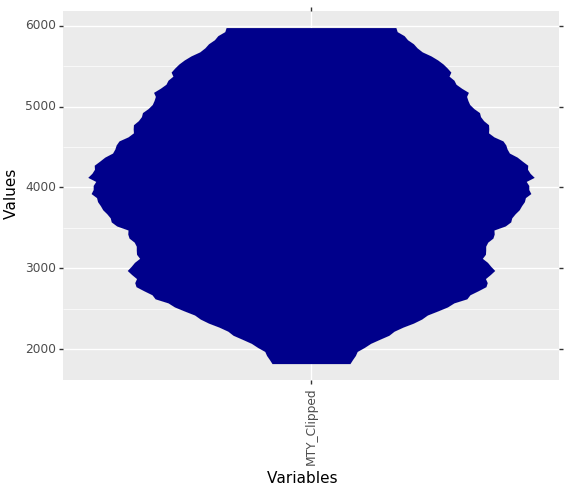


**Fig. 7:** From 9467 to 5000 rows sampled MD-Plot of capped municipality income tax yield of Germany municipalities of 2015.

## 3.3 Scaling

In order to visualize the shapes of all features with very different scales in a plot, the MD-Plot offers four different *Scaling* methods (Percentalize, CompleteRobust, Robust, Log).

- *MDplot(dctExamples['MuncipalIncomeTaxYield_IncomeTaxShare'])*

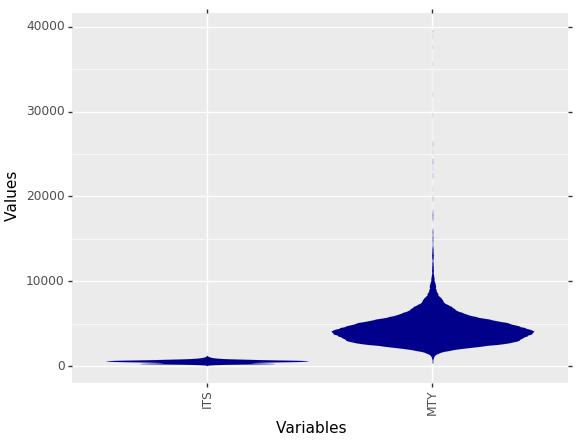


**Fig. 8:** Visualization of two features with different value ranges. The comparison of the distributions is only possible to a limited extent.

- *MDplot(dctExamples['MuncipalIncomeTaxYield_IncomeTaxShare'], Scaling='CompleteRobust')*

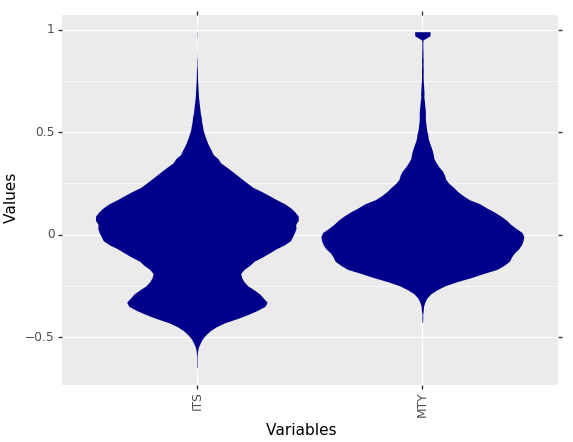


**Fig. 9:** Scaling makes it easy to compare the distributions of features with different ranges of values.

## 3.4 Ordering

The *Ordering* parameter controls the sequence of features displayed. For example, the ordering can be especially useful if one wants to sort the distribution gradually by skewness.

- *dfStocks = dctExamples["StocksData2018Q1"]*
- *dfStocks = dfStocks[["TotalCashFlowFromOperatingActivities", "TreasuryStock", "CapitalExpenditures", "InterestExpense", "NetIncome_y", "NetTangibleAssets", "TotalAssets", "TotalLiabilities", "TotalStockholderEquity", "TotalOperatingExpenses", "GrossProfit", "TotalRevenue"]]*
- *dfStocks = dfStocks[(dfStocks >= -250000) & (dfStocks <= 1000000)]*
- *MDplot(dfStocks, Ordering='Statistics')*

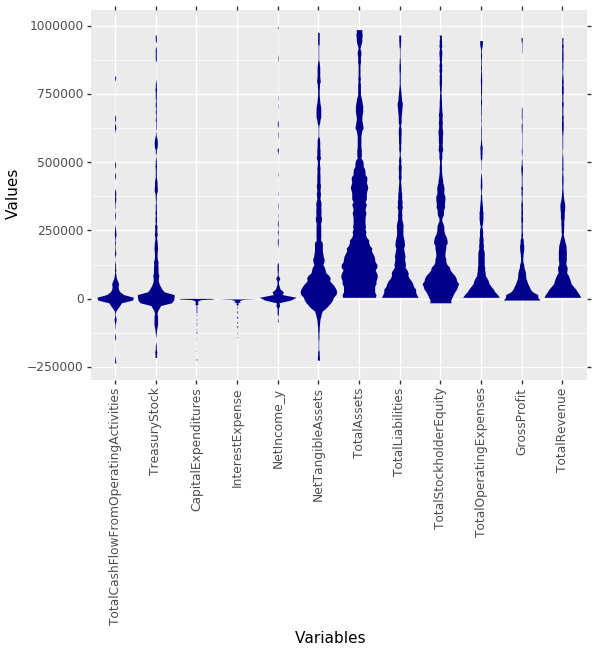


**Fig. 10:** MD plots of selected features from 269 companies on the German stock market reporting quarterly financial statements by the Prime standard. The features are ordered by the effect strength of statistical tests about unimodality and skewness. This leads to an ordering from “Gaussian” features on the left to “Non-Gaussian” features on the right.

- *MDplot(dfStocks, Ordering='Alphabetical')*

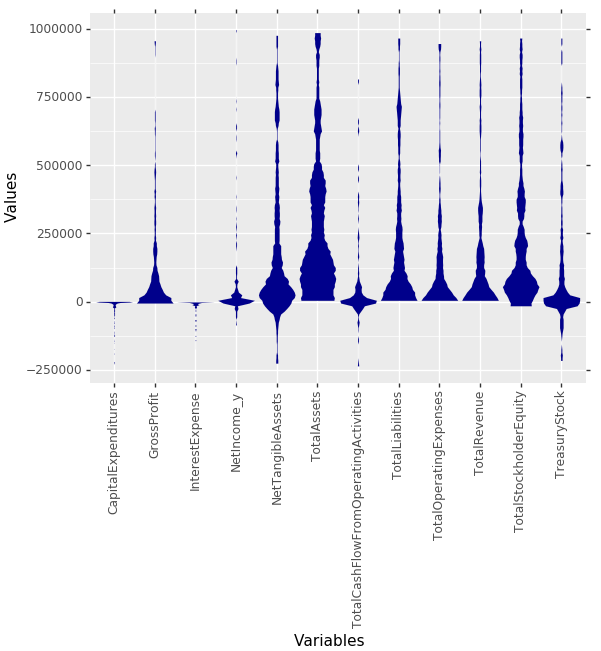


**Fig. 11:** Same stock market features as in Fig. 10, but ordered alphabetical by their name.

# The md_plot package

## 4.1 Availability and Installation

The md_plot package is hosted on the Python Package Index (PyPi) at https://pypi.org/project/md-plot. It can be installed with pip:

pip install md_plot

The source code is available on GitHub at https://github.com/TinoGehlert/md_plot.

## 4.2 Dependencies

The md_plot package is written in pure Python and depends on these packages:

- pandas >= 0.24.2
- numpy >= 1.16.0
- scipy >= 1.1.0
- matplotlib >= 3.1.0
- plotnine >= 0.5.1
- unidip >= 0.1.1

Windows users of Anaconda distribution should update numpy, scipy and matplotlib via conda instead of pip.

## 4.3 Future Improvements

In addition to bug fixing, these improvements to the md_plot package are planned:

- Close the performance gap to the R version
- Reimplementation of dip-test based on diptest R package (unidip is using Monte Carlo simulations to compute the p-values, but this is not the default behavior of the diptest in R)

# References

[Thrun/Ultsch, 2019] **Thrun, M. C., & Ultsch, A.:** Analyzing the Fine Structure of Distributions, Technical Report of the University of Marburg, 2019.

[Thrun et al., 2018] **Thrun, M. C.,Pape, Felix, Hansen-Goos, Onno, & Ultsch A.:** DataVisualizations: Visualizations of High-Dimensional Data, R package available on CRAN**:** <https://CRAN.R-project.org/package=DataVisualizations>, 2018
