# Supplementary material for: Analyzing the fine structure of distributions
Source: PLoS One. 2020 Oct 14;15(10):e0238835. doi: 10.1371/journal.pone.0238835 (PMC7556505; doi:10.1371/journal.pone.0238835)
Supplement: Attachment — Submitted filename: Rebuttal_V2.docx [file pone.0238835.s008.docx]

Dear Editor-in-Chief, Dr. Joerg Heber, Dear Editorial Board,

We are very grateful for the important suggestions written by the first reviewer. However, we are shocked by the quality of the review of the second reviewer and the oversight of the handling editor. This is the first time I ever heard of or read a review in a high-class journal with evidently false statements and no scientific contribution at all. That is not proper handling of a manuscript or reviewing. Due to the anonymity of the second reviewer, we assume competing interests. We hereby report scientific misconduct.

We have addressed false statements as detailed out in the following (our responses below the reviewers’ comments in red letters). We address the positive and helpful scientific review of the first reviewer shortly afterwards. The claims that the first reviewer has written a positive review and the second reviewer either did not read the manuscript or had competing interests become evident.

Since 20.12.19, the manuscript was reinstated and a revision was allowed. Therefore, we take the opportunity to revise the manuscript in accordance with the suggestions of the first reviewer.

Yours Sincerely

Michael Thrun, Tino Gehlert, and Alfred Ultsch

# General Comments:

**Comments to the Author**  1. Is the manuscript technically sound, and do the data support the conclusions?  The manuscript must describe a technically sound piece of scientific research with data that supports the conclusions. Experiments must have been conducted rigorously, with appropriate controls, replication, and sample sizes. The conclusions must be drawn appropriately based on the data presented.

Reviewer #1: Yes

Reviewer #2: Partly

2. Has the statistical analysis been performed appropriately and rigorously?

Reviewer #1: I Don't Know

Reviewer #2: No

3. Have the authors made all data underlying the findings in their manuscript fully available?  The [PLOS Data policy](http://www.plosone.org/static/policies.action#sharing) requires authors to make all data underlying the findings described in their manuscript fully available without restriction, with rare exception (please refer to the Data Availability Statement in the manuscript PDF file). The data should be provided as part of the manuscript or its supporting information, or deposited to a public repository. For example, in addition to summary statistics, the data points behind means, medians and variance measures should be available. If there are restrictions on publicly sharing data—e.g. participant privacy or use of data from a third party—those must be specified.

Reviewer #1: Yes

Reviewer #2: Yes

4. Is the manuscript presented in an intelligible fashion and written in standard English?  PLOS ONE does not copyedit accepted manuscripts, so the language in submitted articles must be clear, correct, and unambiguous. Any typographical or grammatical errors should be corrected at revision, so please note any specific errors here.

Reviewer #1: Yes

Reviewer #2: Yes

# Reviews

Reviewer #2: “This paper studies the mirrored desity (MD) plot and show various structures of the MD results.”

This statement is incorrect. We propose a method for visualization of univariate probability density functions (pdf’s) in data consisting of several features to present the pdf’s in a single visualization. The results show the comparison to the state of the art methods. E.g., this was stated in the abstract of the manuscript:

“Data visualization tools should deliver a sensitive picture of the univariate probability density distribution (PDF) for each feature. Visualization tools for PDFs are typically kernel density estimates and range from the classical histogram to modern tools like bean or violin plots. These visualization tools are evaluated in comparison to statistical tests for the typical challenges of explorative distribution analysis. Conventional methods have difficulties in visualizing the pdf in case of uniform, multimodal, skewed and clipped data if density estimation parameters remain in a default setting. As a consequence, a new visualization tool called Mirrored Density plot (MD plot) is proposed which is particularly designed to discover interesting structures in continuous features”

We also motivated the usage by:

“We compare the visualizations to basic descriptive statistic and show which visualization tools do not visualize the shapes of the pdf accurately. Table 1 summarizes the interesting basic properties from the perspective of data mining and the methods used to compare performance.”

Which with the suggestions of the second reviewer was changed to

“

In sum, the right choice of parameters is interrelated with the properites of data that are unknown in an unsupervised or exploratory data mining task. Table 1 summarizes the interesting basic properties from the perspective of data mining and the methods used to compare performance.

Therefore, we compare the visualizations to basic descriptive statistics and show which visualization tools do not visualize the shapes of the pdf accurately without changing the default parameters of the investigated visualization methods.

“

Table 1: Summary of basic properties of empirical distributions that are interesting for data mining.

| **Interesting basic Properties** | **Exemplary data mining applications** | **Statistical test used** | **Descriptive Statistic** |
| --- | --- | --- | --- |
| Uniformity versus multimodality | Biomedical data (13)  Water vapour (14) | Hartigans’ dip test (6) | Difference between mean and median can indicate multimodality, several coefficients (14) |
| Data clipping versus Heavy-tailedness | Flood data (15), Upper Income (16). | Not required here, but we can refer to (15, 17) | Range of data is sufficient for the task. “There is no easy characteristic for heavy-tailedness” (18) |
| skewness versus normality | Biomedical data (19), Strength of Glass Fibres & Market Value Growth (20) | D'Agostino test(8) | Third order statistics, e.g. (19) |

”

We added in Table 1 Water vapor as further application and exemplary coefficients for bimodality.

This paper proposed a MD plot implemented in Python.

This statement is false. We propose a method which is implemented in R and Python and compare this method with state-of-the-art methods in R and Python. We provide in R a vignette and in Python an introductory tutorial:

Examplary stated in the methods section of the manuscript in 2.1:

“To make sure that the here introduced MD plot does not depend on the specific implementation we provide the package two different programming languages (R and python) reproducing the R results presented in this manuscript below in the python tutorial attached to this work.”

Examplary the reviewed manuscript had the sentences in the methods section 2.3:

“The MD plot can be applied by using the R package ‘DataVisualizations’ on CRAN (21). In the next section, the visual performance of indicating the correct distribution of features is investigated by the histogram, violin, and bean plot in comparison to MD plot. The Python implementation of the MD plot is provided in the Python package ‘md_plot’ on PyPi (22). The vignettes describing the usage and providing the data are attached to this work for the two most-common data science programming languages Pythongand R.

And in the conclusion section:

“The MD plot is available in the R-package ‘DataVisualizations’ on CRAN (21) and in the

Python package ‘md_plot’ on PyPi (22).”

21. Thrun MC, Ultsch A. Effects of the payout system of income taxes to municipalities in Germany.

In: Papież M, Śmiech S, editors. 12th Professor Aleksander Zelias International Conference on

Modelling and Forecasting of Socio-Economic Phenomena; Cracow, Poland: Cracow: Foundation

of the Cracow University of Economics; 2018. p. 533-42.

22. Gehlert T. md_plot: A Python Package for Analyzing the Fine Structure of Distributions. 2019.

Python package.“

Using the insights of the reviewer below, we specified the sentence to

“The MD plot is integrated into the R-package ‘DataVisualizations’ on CRAN (37) in the framework of ggplot2, and in the Python package ‘md_plot’ on PyPi (38)“

Since mirrorred density (MD) plot has been developed in R already, the contribution of this paper is not clearly justified.

This statement is false. One important goal is to show that our algorithm works indepently to the programming language and specific implementation. The authors would like to refer to 2.1:

“Comparing visualizations is challenging because they have the same issues as the estimation of quantiles or clustering algorithms like k-means or Ward: they depend on the specific implementation (c.f. (9), (10),(11, 12)). Therefore, this work restricts the comparison to several conventional methods and specifies the programming language, package and pdf estimation approach used in order to outline several relevant problems for visualization the basic properties of the pdf. To make sure that the here introduced MD plot does not depend on the specific implementation we provide the package two different programming languages (R and python) reproducing the R results presented in this manuscript below in the python tutorial attached to this work.”

The method MD plot was developed for this manuscript by the first author. It is included for technical reasons in the package ‘DataVisualizations’ which has initially been development by the first author and others for various conventional visualization shortcuts and approaches used in Data Science. As every R package on CRAN requires a citation, this package itself is cited with a conference publication about the visual investigation of correlation coefficients. A short look into the description file on CRAN would yield

“Gives access to data visualisation methods that are relevant from the data scientist's point of view. The flagship idea of 'DataVisualizations' is the mirrored density plot (MD-plot) for either classified or non-classified multivariate data presented in Thrun et al. (2019) <[arXiv:1908.06081](https://arxiv.org/abs/1908.06081)>. ”

which clearly references this publication with a preprint published in arXiv as suggested by PLOS ONE. For any user of packages in R or python (i.e. all data scientists) this is obvious.

It is not clear what kind of new features this paper introduce into the MD plot.

As the reviewer evidently did not even read the abstract of the mansucript, it is indisputable that the features are unclear for the reviewer.

Reviewer #1: The paper draws important attention to the pitfalls of existing distributional visualizations for effectively summarizing the nuances of non-normal distributions. Particularly, the paper assesses the efficacy of existing graphical representations (violin plot, box plot, bean plot) for summarizing skewed, multimodal, and uniform distributions, and provides a context and implementation to introduce 'mirror density plots' as an alternative.

The context given for existing visual tools is sound, though there are some areas that could be improved:

We thank the first reviewer for the clear stating that our methodology is sound and problems shown in the paper important.

- Regarding histograms in section 2.2: “…in this work, only default parameter will be used because layman would probably not adjust parameters”. Given that the target user of a statistical visualization package in R or Python likely has experience in data science or statistics, this assumption warrants re-examination. For example: adding (bins=“auto”) is a common procedure for researchers using matplotlib’s built-in histogram function (see: <https://docs.scipy.org/doc/numpy/reference/generated/numpy.histogram_bin_edges.html#numpy.histogram_bin_edges>). Additional context for histograms could be improved with an acknowledgement of non-uniform binning methods, though this may not be in scope for the paper.

We agree with the reviewer that parameters of binning are not the scope of this paper.

Additionally, besides the “auto” parameter in Python, the setting of parameters for the binning of histograms in any exploratory analysis is unfeasible. We elaborated this topic now methods section and changed

“In the last step, we exploratively investigate a new data set with several features with unknown basic properties in order to summarize the challenges of visualizing the estimated probability density function. In such a typical data mining setting, it would be a very challenging task to adjust parameters of the conventional visualizations tools investigated here. We compare the visualizations to basic descriptive statistics and show which visualization tools do not visualize the shapes of the pdf accurately. Table 1 summarizes the interesting basic properties from the perspective of data mining and the methods used to compare performance.”

To

“

In the last step, we exploratively investigate a new data set with several features with unknown basic properties in order to summarize the challenges of visualizing the estimated probability density function. In such a typical data mining setting, it would be a very challenging task to adjust parameters of the conventional visualizations tools. For example, for visualizations of high-dimensional data, either one is unable to set the parameters of a method correctly because the appropriate adjustments are unknown (e.g., p.42, Fig. 5.2 in (9)) or one sets the parameters for a specific dataset correctly in the case that the choice is known beforehand (10). However, then this choice becomes automatically inappropriate for a dataset with unsimilar properties (e.g., p.8 Figure 7, p. (10)). In sum, the right choice of parameters is interrelated with the properites of data that are unknown in an unsupervised or exploratory data mining task. Table 1 summarizes the interesting basic properties from the perspective of data mining and the methods used to compare performance.

Therefore, we compare the visualizations to basic descriptive statistics and show which visualization tools do not visualize the shapes of the pdf accurately without changing the default parameters of the investigated visualization methods.

”

With the suggestion of the reviewer to use ridge line plots, we move the histograms into the supplementary D and instead use ridgelines plots in the manuscript itself.

- It is surprising that this paper contains no ordinary density plots accompanied by sub-axis rugs, which are common methods for analyzing distributions. Similarly, ridgeline plots do not make an appearance, either in discussions of existing visualization methods, or in schematic comparisons of multiple dimensions. Given that these graphical representations seem more common than bean plots, for example, which are discussed at length, background context would benefit from the inclusion of more ordinary non-symmetric representations of distributions.

We thank the reviewer for the suggestion to include ridgelines plots consistently throughout the paper instead of histograms using the ggridges R packages on CRAN, and the ‘kdeplot’ function of the ‘seaborn’ package in supplementary E.

We changed the abstract from

“Visualization tools for PDFs are typically kernel density estimates and range from the classical histogram to modern tools like bean or violin plots.”

To

“Visualization tools for PDFs are typically kernel density estimates and range from the classical histogram to modern tools like ridgeline plots, bean or violin plots.”

We added to the methods section in the case of R

“Another approach consists of ridgeline plots. “Ridgeline plots are partially overlapping line plots that create the impression of a mountain range” (31). They are in R available in the ggridges packages on CRAN (31) and either use the density estimation approaches of R discussed above (if set manually) or per default “estimates the data range and bandwidth for the density estimation from the entire data at once, rather than from each individual group of data” (31). The default setting is used in this work.”

And in the case of Python

“The density plots and ridgeline plots in Python presented in supplementary E are created by using the ‘kdeplot’ function of the ‘seaborn’ package. This approach uses the density estimation by Racine (34) implemented in the ‘statsmodel’ package (35) if it is installed. If it is not installed, the density estimation of ‘scipy’ is used.

“

We added to the results section the parts regarding the ridgeline plot in R and Python and the changed references with regards to the histogram in plotly. For a better overview, we marked these minor changes in this letter using Microsoft Word’s review modus:

“Initially, a random sample of 1000 points of a uniform distribution was drawn and visualized by a commonly usedridgeline plot, violin plot, bean plot, MD plot (Fig. 1) and histogram (SI D, Fig.19) as well as for density estimation in python (SI C, Fig.13, SI E Fig. 24).”

“Fig. 1: Uniform distribution in the interval $[-2,2]$ of a 1000 points sample visualized by a ridgeline plot (a) of ggridges on CRAN (32){Sievert, 2017 #919;Wilke, 2018 #1189}(top) and bottom: violin plot (b, left), bean plot (c, middle) and MD plot (d, right). In the ridgeline plot violin plot and bean plot, the borders of the uniform distribution are skewed contrary to the real amount of values around the borders $2,-2$. The bean plot and ridgeline plot indicate multimodality but Hartigans’ dip statistic (6) disagrees: p(n=1000,D = 0. 01215)= 0.44.”

“Contrary to the expectation, the ridgeline plot, histogram and bean plot indicate multimodality and bean plot, ridgeline plot, and violin plot bend the pdf line in the direction of the end points.”

“Contrary to the expectation, the ridgeline plot, histogram and bean plot indicate multimodality and bean plot, ridgeline plot, and violin plot bend the pdf line in the direction of the end points.”

“This result is visualized in Fig. 3. The bimodality is visible in the ridgeline plot and bean plot starting with a mean equal to $2.4$, and in the MD plot starting with a mean equal to 2.4. However,a robustly estimated Gaussian in magenta is overlaid in the MD plot making bimodality visible starting from a mean of 2.2. Hartigans' dip statistic (6) agrees with these two schematic plots. In contrast, violin plots do not show a bimodal distribution (Fig. 3), while the Python violin and ridgeline plots shows the bimodality starting with a mean equal to 2.4 (SI. C, Fig. 14, SI E, Fig. 25).”

“Fig. 3: Plots of bimodal distribution of changing mean of second Gaussian: Ridgeline plots (a) of ggridges on CRAN (32), violin plot (b), bean plot (c), and MD plot (d). Bimodality is visible beginning from mean 2.4 in bean lot ridgeline plots and MD plot, but the MD plot draws a robustly estimated Gaussian (magenta) if statistical testing is not significant which indicates in mean of two that the distributions is not unimodal. The bimodality of the distribution is not visible in the violin plot”

“Unlike the R version, the skewness is visible in the Python version of the violin plot (SI. C, Fig. 15, SI E, Fig. 26), but slightly less sensitive as the bean plot and MD plot. In the histogram,the skewness of the distribution is difficult to recognize ((SI D, Fig 21),).”

“Fig. 5: Plots of skewed normal distribution by changing the skewness using the R package fGarch (43) on CRAN: Ridgeline plots (a) of ggridges on CRAN (32), violin plot (b), bean plot (c) and MD plot (d). The sample is with n=15000 large. The violin plot is less sensitive for the skewness of the distribution. MD plot allows for an easier detection of skewness by ordering the columns automatically.”

“This issue can also be observed with the Python violin and density plots (SI. C, Fig. 16, SI. E, Fig. 27).”

“In Fig 8, it is visible that the violin plot underestimate skewness of the distribution contrary to the MD plot. The ridgeline, violin and bean plot shows a mode in the skewed distribution between 4 to 4.5 contrary to the MD plot (Fig. 8). In SI D, Fig. 22, the histogram agrees with the MD plot and disagrees with the bean plot that there are no values above 4.35 meaning that the ridgeline and bean plot visualizes a pdf above the maximum value (marked with red lines”

“The Python density and violin plot show, like the bean plot, values above 4.35, but smoothes the distrubition more (SI. C, Fig. 17, SI E, Fig. 28) and, hence, does not indicate multimodality.”

“Fig. 7: Distribution Analyses performed on the log of German people’s income in 2003 with Ridgeline plots (a) of ggridges on CRAN (37) does not indicate clipping or multimodality. (24)”

“In such a high-dimensional case, statistical testing, parameter settings, usual density plots. and histograms become very troublesome and thus are omitted in this work. Moreover, it becomes challenging to integrate different ranges in one visualization. In Tab. 1, SI B, the ordering of the descriptive statistics is from top to bottom is the same as in MD plot, ridgeline and bean plot from left to right. The MD plot enables ordering by concavity which is used here. The MD plot (Fig 9), the bean plot (Fig. 10a) and the ridgeline plot (Fig 10b) visualizes all variables in one picture.”

“The bean plot changes skewed distributions to distributions with one mode or uniform distributions (Fig. 10a). In the bean plot and ridgeline plot (Fig. 10b) there are no hard cut around the value zero (red line).”

“In sum, the visualization of MD plot is in agreement of descriptive statistics (SI B, S1 Table) and disagreement with the bean plot and ridgeline plot. The Python violin and ridgeline plots show values above and below the limits of [-250000, 1000000] and less detailed, and incorrectly unimodal distributions. (SI. C, Fig. 18, SI E, Fig. 29).”

“Fig. 10: Bean plots of selected features from 269 companies on the German stock market reporting quarterly financial statements by the Prime standard (top, a) and Ridgeline plots (b, bottom) of ggridges on CRAN (37). The ordering of the features is by concavity and the same as in Fig. 9. There is no hard cut around the value zero (red line) and the variables are unimodal or uniform with a large variance and a small skewness. The visualizations disagrees with the descriptive statistics in SI B, S1 Table. Note, that for a better comparison we disabled the additional overlaying plots in beanplots.”

“However, the violin plot is unable to visualize the bimodal distribution and the overlayed histogram underestimates it significantly (SI. D, Fig. 23). The Python density and violin plots draw data above and below the limits of the data, but is showing the bimodality of the ITS feature (SI. C, Fig. 19, SI E, Fig. 30).”

In the discussion:

“Three artificial and four natural datasets show the limitations of the schematic plots of ridgeline plot, bean plot, violin plot (R and Python versions). A comparison of results to conventional statistical testing and histograms is included. The results illustrate that the usefulness of the ridgeline plot, violin or so-called bean plot depends on the density estimation approach used in the algorithm, Three artificial and four natural datasets show the limitations of the schematic plots of ridgeline plot, bean plot, violin plot (R and Python versions). A comparison of results to conventional statistical testing and histograms is included. The results illustrate that the usefulness of the ridgeline plot, violin or so-called bean plot depends on the density estimation approach used in the algorithm,…”

“…by changing the skewness parameter (sample size n=15000). Statistical testing, the ridgeline plot, bean plot and MD plot have a similar sensitivity regarding bimodality and skewness as long as the sample is large enough.”

“Automatically ordering the features makes skewness more clearly visible in the MD plot in comparison to the ridgeline plot, bean plot and Python violin plot. The natural example of the Log of German peoples income showed that for smaller samples (n=500) the ridgeline plot, bean plot visualizes unimodal distributions instead of skewed distributions disagreeing with the histogram and MD plot. Additionally, the ridgeline plot, bean plot….”

“with the Python versions. For clipped data, the density estimates of the MD plot does not change contrary to the bean plot.”

“The parameter settings of all plots until the last experiment remained at default because a non-expert user would not change them and an expert user would have difficulties to set density estimation parameters in a solely explorative approach for each feature seperatly. Although the Python ridgeline and the violin plot use density estimators implemented in different packages, both only show marginally different results with the default setting.

”

- The paper would benefit from a ridgeline plot with a single axis, comparing the same non-normal distribution with various binning methods (e.g. each: default histogram using n=10; SciPy's "auto" method mentioned above; Scott's Rule mentioned in paper and detailed on SciPy link above; proposed PDE method; any others potentially relevant according to authors' literature review)

In supplementary E we now state:

“This section covers density plots and ridgeline plots created by using the ‘kdeplot’ function of the ‘seaborn’ package. The default value (Scott's rule of thumb) of the bandwidth parameter was used.”

We want to remark that in exploratory data analysis, the setting of any parameters would become infeasible because the real pdf is unknown, and the parameters would have to be set for each pdf estimation seperatly which cannot be done for many features manually in a limited time.

This is specified in the manuscript in the methods section (see above) and in the discussion:

From

The parameter settings of all plots until the last experiment remained at default because a non-expert user would not change them and an expert user would have difficulties to set density estimation parameters in a solely explorative approach”

To

“The parameter settings of all plots until the last experiment remained at default because a non-expert user would not change them and an expert user would have difficulties to set density estimation parameters in a solely explorative approach for each feature seperatly.”

And in the conclusion it was stated in the manuscript already:

“Adjusting the parameters of conventional plots would require prior knowledge or statistical assumptions about the data, which are often difficult to acquire.”

Additonally, investigating various parameters would overload the manuscript as we alreardy provide 30 figures.

The fundamental scientific contribution of the paper is the usage of the Pareto Density Estimation (PDE) to construct a visualization of a univariate distribution which captures non-normal characteristics of distributions, such as skew, multimodality, and uniformity. This method appears well-supported, and is explained concisely, with easily accessible packages for both R and Python to supplement the work.

Thank you for getting the concept of our paper and understanding the usual references of the packages.

While the PDE method for binning appears well-defended, the implementation into a visual language leaves some questions unanswered:

- In the broader data visualization community, "Mirror Density" plots are bivariate distributions: for example, one might construct two violin plots of distributions, conditioned on a second binary value (e.g. control vs. experiment), split the resultant forms in half lengthwise, and position them opposite one another to create a comparative representation of the conditioned distributions (see: <https://www.d3-graph-gallery.com/graph/density_mirror.html>). In this bivariate application, the comparative symmetry adds value to the analytic process. It is unclear from the paper whether the authors are aware of this namespace convergence, but independent of nomenclature, the paper would benefit from an assessment of the analytic value for making a univariate density plot symmetric.

Of this ambiguous naming of plots, we were not aware. We discuss this ambivalence in the corrected version of the manuscript in the methods section

“It should be noted that there exists an ambiguity in the naming because of the existence of “Mirror Density” plots, a graphical representation of bivariate distributions (e.g., https://www.d3-graph-gallery.com/graph/density_mirror.html). However, maximum likelihood plots can be more informative for such a use case (e.g. (39)). In the opinion of the authors, the name “Mirrored Density” plot (MD plot) is more specific than the “Mirror Density” plot because the density estimation is univariate with a graphical representation as a “line” with a symmetrical reflection of the same information filled out with a color instead of using a bivariate density estimation which does not mirror the “line” of the plot.

- In section 3.5 "The high-dimensional data set (d=45)... is investigated by selecting 12 features": Ridgeline plots with the PDE binning method may be a more space-conservative method of implementing the algorithm (though admittedly, d=45 remains a non-trivial 'curse of dimensionality').

We tried this approach, but for too many features, it becomes infeasible to inspect such a plot. We think that to outline this point is out of the scope of the manuscript.

- With regard to the German stock market data in section 3.5, the schematic MD (Fig. 9) and violin (Fig. 10) plots compare distributions in very different ranges. The paper would benefit from the removal of 'InterestExpense' and 'CapitalExpenditures' from the exemplary features, perhaps to be replaced with features of a range more similar to the other features in the schematic plots.

We thank the reviewer for pointing out this issue. We now are aware of specifying our work more. We added to the results section

“In such a high-dimensional case, statistical testing, parameter settings, usual density plots. and histograms become very troublesome and thus are omitted in this work. Moreover, it becomes challenging to integrate different ranges in one visualization.”

- In general, plots should be ordered where possible, e.g. Fig. 5b,c,d should show skew parameter xi in order [0.6,0.95,1,1.1] for clarity.

The authors thank the reviewer for the suggestion. The mistake was corrected.

- Stacked histograms are not advisable for this application. Stacked histograms make sense when considering how categories sum to a total population (e.g. when exploring various revenue sources and the resultant aggregate revenue in a single graphic). For comparing model distributions of various skewness parameters, e.g. in Fig. 3a, 5a (are these the same histogram?), stacking does not seem appropriate. Neither does stacking seem appropriate for histograms of normalized data, e.g. Robustly Normalized values for Income Tax Share (ITS) and Municipality Income Tax Yield (MTY) in Fig. 12a. The sum total of 2 bins normalized from different ranges provides no substantial comparative analytic value. In both stacked histogram cases: overlaying, rather than stacking, may provide the intended visual effect, and would be appropriate to the data.

The authors thank the reviewer for pointing out this major issue. The manuscript is now rewritten focusing more on ridgeline plots instead of histograms. Histograms are moved to supplementary D because they are challenging to use if many features are given, and the (in)correct binning issue can always be raced.

The authors thank the reviewer for pointing out the correct definition for stacked histograms. The wording was changed throught the manuscript from “stacked” to “overlayed” because stacked histograms were not computed in the definition of the reviewer above. To be more precise supplementary D has now the sentence:

“Each histogram is computed seperatly and thereafter integrated in one plot using plotly.”

The authors did a mistake by providing both times an overlayed histogram for the skewed experiment instead providing an overlayed histogram for the skewed distribution and one for the bimodal experiment. This is now also corrected.

As a reviewer with experience in data visualization, I feel confident in my assessment of this component of the paper; however, it is my hope that fellow peer reviewers can speak in a more informed manner on the statistical evaluations and experiments performed. The paper's conclusion in Section 5 "current density estimation approaches can lead to major misinterpretations if the default setting is not adjusted" seems to suggest that the scientific community would benefit greatly from the addition of PDE binning methods to existing open-source visualization packages such as ggplot, matplotlib, seaborn and plotly. I hope the authors consider integrated contribution to existing open-source tools.

MD plot was integrated in the ggplot2 syntax and specified in the methods section

“The MD plot can be applied by using installing the R package ‘DataVisualizations’ on CRAN (36) in the framework of ggplot2 (39).”

And in the conclusion section we changed

“The MD plot is available in the R-package ‘DataVisualizations’ on CRAN (33) and in the Python package ‘md_plot’ on PyPi (34). “

To

“The MD plot is available integrated into the R-package ‘DataVisualizations’ on CRAN (36) in the framework of ggplot2, and in the Python package ‘md_plot’ on PyPi (37).

“

Our co-author will try to contact seaborn regarding this idea if our work gets published in a peer-review journal.

The paper would be improved by a spelling check, and a grammar proofread by a native English speaker. Typos and grammatical oversights do not obstruct communication, but do inhibit narrative flow.

The non-native authors are terribly sorry for the inconvenience. We now applied Google’s Grammarly for spell checking. If accepted, Springer Nature could be payed for grammatical corrections and spell-checking.

The Reviewers lead to the following decision by the handling editor Qichun Zhang:

“

PONE-D-19-19081

Analyzing the Fine Structure of Distributions

PLOS ONE

Dear Dr. rer. nat. Thrun,

Thank you for submitting your manuscript to PLOS ONE. After careful consideration, we have decided that your manuscript does not meet our criteria for publication and must therefore be rejected.

I am sorry that we cannot be more positive on this occasion, but hope that you appreciate the reasons for this decision.

Yours sincerely,

Qichun Zhang, PhD

Academic Editor

PLOS ONE

Additional Editor Comments (if provided):

Two reviewers returned the critical comments focusing on the novelty of the manuscript. Basically, the author redo some existing method using Python where the new features have not been demonstrated clearly. In addition, the English writing should be pre-checked where some typos would affect the readability of the manuscript.

“

The authors have shown in this letter that

- the first reviewer positively reviewed our manuscript, and all suggestions of this reviewer were applied throughout the mansuscript
- the second reviewer, as well as apparently the handling editor, did not read the manuscript at all.

It seems that the handling editor just performed a copy and paste action of the evidently false statements of the second reviewer, ignoring the first reviewer's comments except for the last comment regarding the spelling.

To find out that the statements are false would take less than an hour of work. Any editor should at least be curious about a review consisting of four sentences after a long waiting period of 5 months and check the claims of such a review.

Additionally, minor spelling and grammatical errors should not influence the decision of an editor in a scientific journal at all.
