# Supplementary material for: Analyzing the fine structure of distributions
Source: PLoS One. 2020 Oct 14;15(10):e0238835. doi: 10.1371/journal.pone.0238835 (PMC7556505; doi:10.1371/journal.pone.0238835)
Supplement: Attachment — Submitted filename: Response to Reviewers 1.docx [file pone.0238835.s009.docx]

Dear Editor Dr Fatemeh Vafaee,

thank you for putting the large effort into improving our manuscript entitled “Analyzing the Fine Structure of Distributions” and giving us the chance to modify it in order to accommodate the new reviewer’s comments.

We have addressed the comments as detailed out in the following (our responses below the reviewers’ comments in red letters).

PONE-D-19-19081R2

Analyzing the Fine Structure of Distributions

PLOS ONE

Dear Dr. Thrun,

Thank you for submitting your manuscript to PLOS ONE. After careful consideration, we feel that it has merit but does not fully meet PLOS ONE’s publication criteria as it currently stands. We apologize for the delay in getting back to you as we had difficulty in finding enough reviewers for the revised version of your manuscript. Not all the initial reviewers were available to review the revised version and finding a reviewer with relevant expertise who would accept to review has taken a long time.

Nonetheless, we invite you to submit a revised version of the manuscript that addresses the points raised by Reviewer #5. Please submit your revised manuscript by Aug 28 2020 11:59PM. If you will need more time than this to complete your revisions, please reply to this message or contact the journal office at [plosone@plos.org](mailto:plosone@plos.org). When you're ready to submit your revision, log on to <https://www.editorialmanager.com/pone/> and select the 'Submissions Needing Revision' folder to locate your manuscript file.

Please include the following items when submitting your revised manuscript:

- A rebuttal letter that responds to each point raised by the academic editor and reviewer(s). You should upload this letter as a separate file labeled 'Response to Reviewers'.
- A marked-up copy of your manuscript that highlights changes made to the original version. You should upload this as a separate file labeled 'Revised Manuscript with Track Changes'.
- An unmarked version of your revised paper without tracked changes. You should upload this as a separate file labeled 'Manuscript'.

If you would like to make changes to your financial disclosure, please include your updated statement in your cover letter. Guidelines for resubmitting your figure files are available below the reviewer comments at the end of this letter.

If applicable, we recommend that you deposit your laboratory protocols in [protocols.io](http://protocols.io/) to enhance the reproducibility of your results. [Protocols.io](http://protocols.io/) assigns your protocol its own identifier (DOI) so that it can be cited independently in the future. For instructions see: <http://journals.plos.org/plosone/s/submission-guidelines#loc-laboratory-protocols>

We look forward to receiving your revised manuscript.

Kind regards,

Fatemeh Vafaee, Ph.D.

Academic Editor

PLOS ONE

[Note: HTML markup is below. Please do not edit.]

Reviewers' comments:

Reviewer's Responses to Questions

**Comments to the Author**  1. If the authors have adequately addressed your comments raised in a previous round of review and you feel that this manuscript is now acceptable for publication, you may indicate that here to bypass the “Comments to the Author” section, enter your conflict of interest statement in the “Confidential to Editor” section, and submit your "Accept" recommendation.

Reviewer #4: All comments have been addressed

Reviewer #5: (No Response)

2. Is the manuscript technically sound, and do the data support the conclusions?  The manuscript must describe a technically sound piece of scientific research with data that supports the conclusions. Experiments must have been conducted rigorously, with appropriate controls, replication, and sample sizes. The conclusions must be drawn appropriately based on the data presented.

Reviewer #4: Yes

Reviewer #5: Partly

3. Has the statistical analysis been performed appropriately and rigorously?

Reviewer #4: Yes

Reviewer #5: N/A

4. Have the authors made all data underlying the findings in their manuscript fully available?  The [PLOS Data policy](http://www.plosone.org/static/policies.action#sharing) requires authors to make all data underlying the findings described in their manuscript fully available without restriction, with rare exception (please refer to the Data Availability Statement in the manuscript PDF file). The data should be provided as part of the manuscript or its supporting information, or deposited to a public repository. For example, in addition to summary statistics, the data points behind means, medians and variance measures should be available. If there are restrictions on publicly sharing data—e.g. participant privacy or use of data from a third party—those must be specified.

Reviewer #4: Yes

Reviewer #5: Yes

5. Is the manuscript presented in an intelligible fashion and written in standard English?  PLOS ONE does not copyedit accepted manuscripts, so the language in submitted articles must be clear, correct, and unambiguous. Any typographical or grammatical errors should be corrected at revision, so please note any specific errors here.

Reviewer #4: Yes

Reviewer #5: Yes

6. Review Comments to the Author  Please use the space provided to explain your answers to the questions above. You may also include additional comments for the author, including concerns about dual publication, research ethics, or publication ethics. (Please upload your review as an attachment if it exceeds 20,000 characters)

Reviewer #4: (No Response)

Reviewer #5: The authors present a variant of the violin plot, termed “mirrored density” plot, which is intended to provide users with a more useful depiction of the underlying univariate distribution for the purposes of data exploration. The authors correctly highlight the fact that the default parameters for many popular packages may not be suitable for data exploration purposes as they are not sensitive enough to the fine structure of the data. The mirrored density plot is proposed to address this shortcoming of existing visualization software by utilizing Pareto Density Estimation for the estimation of univariate probability densities.

In order to argue for the adoption of mirrored density plots, the authors present a series of experiments on both simulated and real datasets in which mirrored density plots are compared to violin, ridgeline, and bean plots. Statistical tests were performed on simulated datasets to test for the presence of certain assumed/designed features (i.e bimodality and/or skewness), and plots were qualitatively inspected for agreement with these tests.

I commend the authors for reproducing their work in Python in addition to R to ensure that performance is not implementation dependent. Reproducibility is in important concern, and it is good to see the authors taking measures to ensure consistency.

There are several issues that I feel need to be addressed:

1) It is unclear why vioplot was used as the representative package for violin plots. ggplot2 is more widely used and accepted within the R community (155K monthly downloads vs 9K – although ggplot does a lot more than violin plots to be fair).

The authors would like to thank the reviewer for his effort in reviewing this work. In the last draft of the manuscript, it was stated:

“

The violin plot uses a nonparametric density estimation based on a smooth kernel function with a fixed global radius [33]. The R package ‘vioplot’ on CRAN [34] serves as a representative for this work using the density estimation with the bandwidth defined by a Gaussian variance of the R package 'sm' on CRAN [35]. An alternative approach that is commonly applied is to use the density estimation of the R package 'stats' [36], where the bandwidth is usually computed by estimating the mean integrated square error [37], but several approaches can be chosen.”

”

The method geom_violin is also referenced with [4] which means that no scientific improvements were made. Geom_violin uses the density estimation of [36] with a different parameter setting to the violin plot of vioplot. Having already investigated many methods in two languages, we did find it unnecessary to add another method.

We extended the methods section with two additional sentences and specified:

“The violin plot [4] uses a nonparametric density estimation based on a smooth kernel function with a fixed global radius [33] (…) chosen. As an alternative, the geom_violin method [4] of the well-known “ggplot2” package [47] is presented in SI F, which uses the density estimation specified in [36].

Furthermore, the underlying functionality for MDplot is provided by ggplot2’s violin plot (<https://github.com/Mthrun/DataVisualizations/blob/bc76a8c6dc737cb5c593479a534ef2a5b60b330e/R/ClassMDplot.R#L148>), so it seems strange not to use this package for comparison.

We are grateful to the reviewer in helping us in improving our documentation. ClassMDplot is now correctly referenced with an published abstract:

Thrun, M. C., Breuer, L., & Ultsch, A. : Knowledge discovery from low-frequency stream nitrate concentrations: hydrology and biology contributions, Proc. European Conference on Data Analysis (ECDA), Paderborn, Germany, 2018.

indicating that this research is ongoing and not the focus of this manuscript.

The correct point of reference is

https://github.com/Mthrun/DataVisualizations/blob/6ab2d66c1eebdb8e3b36529bb2b9b254cf44b364/R/MDplot.R#L455

it is showing clearly that this call is a small part of a large algorithm.

This is the reason we stated in the last draft of the manuscript:

“The MD plot can be applied by installing the R package ‘DataVisualizations’ on CRAN [44] in the framework of ggplot2 [47]. The Python implementation of the MD plot is provided in the Python package ‘md_plot’ on PyPi [45]. ”

which means that the geom_violin call is not necessary (see python) but hopefully more convenient for R user.

Please see Replication_Exp1_Fig3.svg. This figure shows the mirrored density plot overlayed with two violin plots from ggplot2. The green outline was produced with default parameters, and the red line with the minor adjustments which will be described below. When using ggplot2’s violin plot, multimodality is clearly visible when the second mean is 2.4 or 2.5 unlike the plots produced by vioplot.

We are very grateful for allowing us the chance to improve our manuscript. In the last draft it was stated:

“

For example, when visualizing high-dimensional data, one is unable to set the parameters of a method correctly because the appropriate adjustments are unknown (e.g., p.42, Fig. 5.2 in [14]) or one sets the parameters for a specific dataset correctly because the choice is known beforehand [15]. However, this choice automatically becomes inappropriate for a dataset with unsimilar properties (e.g., p.8, Fig. 7, p. [15]). In sum, the right choice of parameters is interrelated with the properties of data that are unknown in an unsupervised or exploratory data mining task.

“

and

“

The parameter settings of all plots until the last experiment remained at default because a nonexpert user would not change them and an expert user would have difficulties setting density estimation parameters in a solely explorative approach for each feature separately.

”

These parts are now extended to:

“

For example, when visualizing high-dimensional data, one is unable to set the parameters of a method correctly because the appropriate adjustments are unknown (for example p.42, Fig. 5.2 in [14]) or one sets the parameters for a specific dataset correctly because the choice is known beforehand [15]. However, this choice automatically becomes inappropriate for a dataset with unsimilar properties (for example p.8, Fig. 7, p. [15], see also the example in SI F (4.)). In sum, the right choice of parameters is interrelated with the properties of data that are unknown in an unsupervised or exploratory data mining task.

“

“..feature separately. The effects of tuning parameters are presented exemplary for the ggplot2 method geom_violin in SI F (4.). Certainly, many methods can be tuned to obtain a correct result for a specific distribution if prior knowledge is used. However, the example outlines that tuning parameters for one distribution results in the incorrect visualization for another distribution.”

Please see Replication_Uniform_Fig1.svg. This is a replication of the 1000 uniform samples figure. Again, 2 violin plots are presented on top of the MD plot. The green line is with default parameters. The red line, which almost exactly matches the MD plot, uses kernel=’rectangular’, and adjust=’0.8’. I understand that there is an argument for providing useful default parameters, but I am not convinced it warrants an entirely new package.

We are terribly sorry that the reference figures were not included in the review. We assume a technical error. To fasten the process of review, we decided make this technical argumentation more transparent. Supplementary F is now presented as an R markdown script with data available upon request per email (for datasets not already provided in DataVisualizations). We hope that the argumentation taken here is now more clear.

The use of vioplot instead of ggplot2 is largely responsible for the author’s claim that “Violin plots in R were not able to visualize the bimodality, which was surprising.”

The different choice of parameter settings is responsible for this fact, which does not contradict our main findings. Nevertheless, the following passages are now changed

“In contrast, violin plots do not show a bimodal distribution (Fig. 3),…”

To

“In contrast, except for geom_violin of ggplot2 (see SI F), the violin plots examined here do not show a bimodal distribution (Fig. 3),..”

“Fig3. …. The bimodality of the distribution is not visible in the violin plot.”

To

“Fig3. …. The bimodality of the distribution is not visible in the violin plot [4] of the implementation [34].”

“

Violin plots in R were not able to visualize the bimodality, which was surprising.

”

To

“Violin plots in R were strongly depending on specific parameter settings in order to visualize the bimodality only in cases that it existed, which was surprising.

“

2) “Statistical testing indicated that the ridgeline plot, bean plot, and MD plot have a similar sensitivity regarding bimodality and skewness as long as the sample is large enough.” – This is a gross misrepresentation of the statistical testing performed in this work. The statistical tests referred to were intended to test for the presence of bi-modality or skewness in the simulated datasets. They do not assess the performance of plotting methods. As such, this should not be taken as statistical evidence supporting MDplot’s performance. This paper is ultimately a qualitative comparison of methods and should be treated as such.

We thank the reviewer for the chance to specify the intended meaning of the sentence as follows:

“

The minimal quality requirement to schematic plots is the necessity that the visualizations at least produce comparable results to (“are as sensitive as”) statistical testing and descriptive statistics. In this sense, the comparison of performance showed that the ridgeline, bean, ggplot2’s violin, and MD plot have a similar sensitivity in comparison to ststistics for bimodality and skewness as long as the sample is large enough (Fig. 2-5) but MD plot outperforms for smaller sample sizes (see Fig 1., Fig. 9, and SI B Table 2).

”

3) There is no justification/discussion regarding sample sizes in the simulated datasets which seem to have been chosen arbitrarily. Why were 1000 samples included for the uniform example, 15500 for multimodality, and 15000 for skewness?

We are grateful for the hint allowing us to point our train of thought.

The last draft stated

“Thus, the basic properties of the investigated distributions are well defined as long as the sample size does not become too small.”

The manuscript elaborates now

“Thus, the basic properties of the investigated distributions are well defined as long as the sample size does not become too small. Sample sizes for the artificial datasets in the case of skewness and bimodality are chosen as the maximum size that allows for exact statistical testing. The lower limit is chosen with the artificial dataset of the uniform distribution for which a QQ plot against the uniform distribution would indicate a straight line.”

It would also be valuable to see how each method performs at various sample-sizes as not all data exploration takes place with such a large sample size.

We added as the next sentence:

“…straight line. The here investigated sample sizes for natural and artificial datasets range from 269 to 31.000. The implicit assumption of this work is that for this range of sample sizes, it is not probable that the results of the compared methods will change. In the case of the MD plot, the underlying Pareto density estimation is well-investigated for varying sample sizes [11].”

The smaller/real dataset experiments do not address this question as the “ground-truth” behind the structure is ultimately unknown.

The last draft of the manuscript defined the ground truth as the basic statistical properties:

“Table 1 summarizes the interesting basic properties from the perspective of data mining and the methods used to compare the performance of different methods.

Therefore, we compare the visualizations to basic descriptive statistics and show which visualization tools do not visualize the shapes of the PDF accurately without changing the default parameters of the investigated visualization methods.

“

However, exact specification of ground truth using QQ plots and various other methods was required in order to account for the variance of data and perform standardization of the features. This is now stated in the next sentence:

“Table 1 summarizes the interesting basic properties from the perspective of data mining and the methods used to compare the performance of different methods. Extensive knowledge discovery for this dataset was performed in [17]. (…) methods.”

[17]: [Thrun, 2019] Thrun, M. C.: Knowledge Discovery in Quarterly Financial Data of Stocks Based on the Prime Standard using a Hybrid of a Swarm with SOM, in Verleysen, M. (Ed.), European Symposium on Artificial Neural Networks, Computational Intelligence and Machine Learning (ESANN), Vol. 27, pp. 397-402, Ciaco, 978-287-587-065-0, Bruges, Belgium, 2019.

4) There is no discussion surrounding limitations/shortcomings of the work. It is important to provide this information for potential users so they can make a well-informed decision about whether this package is appropriate for their data. I strongly recommend a discussion surrounding the shortcomings of the qualitative nature of this work.

We are very grateful for this essential suggestion to discuss the limitation of the MD plot. The discussion was extended to

“

The general performance of MD plot seems to be sufficient for data set sizes up to 10^5. Pareto density estimation and, subsequently, the Pareto radius has to be computed for each feature separately which increases the computation time accordingly. Therefore, a parallel implementation of the density estimation is planned in the next iteration. Above 10^5, Pareto density estimation becomes computationally intensive. For big data sets (>10^5) MD plot uses per default an appropriate subsampling method. PDE was not investigated below a sample size of 50 [11]. Thus, below this threshold, no density estimation is performed in the default setting. Instead, a 1D scatter plot with jittered points is drawn.

“

Quantitative comparisons are possible for this sort of work – for example, blind-surveys could be conducted to see whether individuals can detect underlying structure from the plots alone (or whether they detect structure which is not there).

We thank the reviewer for the suggestion and extended the conclusion:

“In future research, a blind survey should be conducted to investigage how well laymen can detect all underlying structures from the MD plots alone.”

Furthermore, there is no discussion surrounding the tendency of this method to over-fit to the data.

We are very grateful for this crucial suggestion. The discussion was extended to

“It should be noted that the Pareto density estimation (PDE), which is used in the MD plot, is specially designed for the detection of multimodality, which could result in an overestimation of multimodality. Such an overestimation would be visible in the “roughness” of the mirrored density of a feature.

“

Minor corrections:

• Throughout the manuscript, both in-text and in-figures, when referring to a normal distribution, m and sd should be replaced with µ and σ respectively (e.g Fig 3b).

We would like to be consistent with prior publications. We elaborated

“

The sample consisting of the first Gaussian N (m=0, sd=1) remained unchanged, and the second Gaussian N (m=i, sd=1) changed its mean through a range of values.

”

To

“

The sample consisting of the first Gaussian N (m=0, s=1) remained unchanged (for definition of Gaussian mixtures please see [50]), and the second Gaussian N (m=i, s=1) changed its mean through a range of values.

”

• The authors should attempt to install their package (DataVisualizations) on a clean installation of R. It does not properly install the required packages. These packages have to be added manually.

The package does not have any warnings or errors on CRAN

<https://cran.r-project.org/web/checks/check_results_DataVisualizations.html>

which tests the example specified for the MDplot meaning that the package is working well on Linux, Solaris, Windows and Mac Os.

Nevertheless we tested this issue manually and we assume that the reviewer called

install.packages("DataVisualizations")

instead of

install.packages("DataVisualizations",dependencies = TRUE)

• It would be nice to have figures either superimposing the MD/violin/bean plots or showing them side by side for an easy visual comparison.

We do not understand this style hint the reviewer suggested. However, as it is not a scientific suggestion, we did not apply changes to the figures. Maybe the figure position could be improved by the editorial office after acceptance.

• When referring to the Skewed normal distribution, you should use SN, and not N, to avoid confusion with the actual normal distribution (e.g fig 5b).

We added the following sentence to the results section to avoid confusion:

“

The case of N(m=0,s=1,xi=1) defines the uniform Gaussian distribution (for definition of Gaussian please see [50], skewed distributions [51]).

”

• Plots should be formatted consistently. For example, titles of some are bolded (5b) while the others are not (5a).

We are terribly sorry that we lack the programming skills to precisely format figures in two programming languages for several technical plotting concepts. Maybe the figure could be post-processed by the editorial office after acceptance. Otherwise we could invest in the post processing software Adobe Photoshop for the proofs if the editor finds this change necessary.

• Fig 6a uses the naming “beanplot” whereas the rest of the paper uses “bean plot”

Thank you for the hint, we will write “bean plot” uniformly in all relevant figures in the case of acceptance.

• In “Given a feature in the data space, there are several approaches for evaluating univariate structures using the indications of the quantity and range of values, e.g., quantile-quantile plots” – e.g. should just be spelled out as “for example”

All “e.g.,” are now spelled out “for example”.

• “The counterparts of the box plot are the range bar [7], and its extension to the notched box plot [8] is nearly unable to visualize multimodality [3]; therefore, it is disregarded in this work. ” – I think you mean to say “The boxplot and it’s counterparts (i.e range bar and notched box) are unable to visualize multimodality and are therefore disregarded in this work.”, but I’m not sure.

This sentence is now corrected to

“

The box plot and it’s counterpart the range bar [7], as well as its extension, the notched box plot [8] are nearly unable to visualize multimodality [3] and are therefore disregarded in this work..

”

• The following quote is missing and ending quote: “Pareto density estimation (PDE), the radius for hypersphere density estimation is chosen optimally w.r.t information theoretic ideas [28].

This was corrected.

• W.r.t (with respect to) above should probably be spelled out for clarity. Square brackets can be used to indicate a quote has been altered.

This was corrected.

• There are several places where the phrase “ridgeline plot, violin plot and bean plot” is used. I would suggest changing to “ridgeline, violin, and bean plots” for brevity. Should this suggestion be ignored, then “Although the Python ridgeline and the violin plot use density estimators implemented in different packages” should be made consistent

This was corrected.

• Plot in the quote above should be plots – same correction applies to “in contrast to the histogram and MD plot.”

This was corrected.

• Remove the first comma in “The results show that the MD plot is the only schematic plot, which is appropriate for every case and does not require adjustments to its process of density estimation by various parameters” (currently it reads as the MD is the only schematic plot, which is isn’t).

This was corrected.

• In “Using web scraping, the information of n=269 cases was extracted.” replace was with were.

This was corrected.

Overall, the English is good, however, there are minor typos and punctuation mistakes scattered throughout. I acknowledge that this was professionally vetted by nature/springer for language editing, but they missed several mistakes.

We thank the reviewer for the hint. Further improvements can be performed in the proofs if the manuscript gets accepted.

Further notes regarding the similarity of “rectangle” kernel estimation to the Pareto Density Estimation approach:

• I have included another comparison of violin and md plots applied to uniformly sampled data (small_uniform_sample.svg). Again, I was able to get quite a similar result using the “rectangle” option for kernel density estimation.

• In all fairness, this required a smaller bandwidth factor (adjust was set to 0.5 instead of 0.8).

• This suggests that there may be an argument to be made in support of PDE as it does a better job showing the fine-grained structure of the data.

Regarding various parameter settings, we refer to the argumentation above. We are sorry that we did not receive the mentioned figures.

• Furthermore, I concede that these plots taper off towards the end which may be misleading to end users.

We again thank the reviewer for the outstanding work that she/he did in reviewing the manuscript and even applying the method and other comparable methods to data. Detailed and transparent evaluation of ggplot2::geom_violin can be now found in supplementary information F saved as an R markdown script.

[NOTE: If reviewer comments were submitted as an attachment file, they will be attached to this email and accessible via the submission site. Please log into your account, locate the manuscript record, and check for the action link "View Attachments". If this link does not appear, there are no attachment files.]

No attachments were found but the text was sufficient to understand the valid points the reviewer made.

While revising your submission, please upload your figure files to the Preflight Analysis and Conversion Engine (PACE) digital diagnostic tool, <https://pacev2.apexcovantage.com/>. PACE helps ensure that figures meet PLOS requirements. To use PACE, you must first register as a user. Registration is free. Then, login and navigate to the UPLOAD tab, where you will find detailed instructions on how to use the tool. If you encounter any issues or have any questions when using PACE, please email PLOS at [figures@plos.org](mailto:figures@plos.org). Please note that Supporting Information files do not need this step.

For the case that in the next round of revision another new reviewer is assigned, which has other suggestions regarding figure style, position, etc., no figures of the last draft were changed besides Figure 31 which was deleted because it is now presented in a broader context in the new supplementary F.

We would like to note that this project has received and receives no funding at all meaning that the whole project is performed in our off-time (vacations, evenings, weekends). The process of generating 62 figure files, testing them in PACE, improving them for PACE, storing them separately from the manuscript, naming them again in the submission system and checking the build pdf of the submission system if no referencing errors were made, is unfeasible without funding and highly time-consuming for the first author. However, figure style changes do not improve the manuscript from the scientific perspective. We wish to remark that we hope that the guidelines allow for storing the figures in the main manuscript in the future - at least during the review and revision process. The additional advantage would be that a manuscript can be easier reviewed. If the scientific discussion ends positively, of course, improvements with regard to the figures can be made in the proofs.

We hope that the manuscript now meets the criteria for publication in PLOS ONE. We are very much looking forward to hearing from you.

Yours sincerely

Michael Thrun, Tino Gehlert and Alfred Ultsch.
